# Experimental Evidence of Peer Gender Nonconformity Triggering Dehumanization in Children: Developmental Trajectory, Form, and Link to Bullying

**DOI:** 10.1111/desc.70070

**Published:** 2025-09-05

**Authors:** Marshall M. C. Hui, Karson T. F. Kung

**Affiliations:** ^1^ Department of Psychology University of Hong Kong Pokfulam Hong Kong Special Administrative Region

**Keywords:** bullying, dehumanization, gender nonconformity, intergroup bias, mind perception, social cognition

## Abstract

Gender nonconforming (GN) children are at higher risk of experiencing bullying and social exclusion than are gender conforming (GC) children. Nonetheless, very little is known about the socio‐cognitive mechanisms underlying children's bias against GN peers. The present study was the first to examine children's dehumanization of GN peers (developmental trajectory, form, and link to bullying). Chinese children in Hong Kong (*N* = 472; in four age groups: 5–6, 7–8, 9–10, and 11–12 years) were assessed individually in an in‐person experimental study. Children were shown experimentally manipulated vignettes of four hypothetical peers (GC boy, GC girl, GN boy, and GN girl). Next, children's blatant and subtle dehumanization of these peers, and their propensity to bully these peers, were assessed. In the blatant dehumanization task, children rated how human‐like/insect‐like each peer was using a visual scale with a continuous slider. For subtle dehumanization, a mind perception task was used to assess the frequency and diversity of mental state words spontaneously used by children to describe videos associated with different peers. Children also completed questions assessing bullying tendency. By age 9–10 years, children dehumanized GN peers both blatantly and subtly, regardless of peer gender. Older children rated GN peers as less human‐like/more insect‐like than GC peers and also spontaneously attributed fewer and less diverse mental states to GN peers than to GC peers. According to multilevel moderated mediation analyses, blatant dehumanization partially explained older children's tendency to bully GN peers. Further research may develop interventions aimed at reducing children's dehumanization of GN peers.

**Summary:**

- 5‐ to 12‐year‐old children's blatant and subtle dehumanization of gender nonconforming peers were assessed.
- Older children (7‐ to 12‐year‐old) blatantly rated gender nonconforming peers as less human‐like/more insect‐like than gender conforming peers.
- Older children (9‐ to 12‐year‐old) spontaneously ascribed fewer and less diverse mental states to gender nonconforming peers than to gender conforming peers.
- Older children's (9‐ to 12‐year‐old) blatant humanness ratings partially explained their propensity to bully gender nonconforming peers.

## 1 Introduction

### 1.1 Gender Nonconformity in Children

Starting from early childhood, children expect their peers to follow gender norms and show preferences for gender conforming (GC) over gender nonconforming (GN) peers and behaviors (Martin et al. 2013; Riggs et al. 2023; Skočajić et al. 2020; Zucker et al. 1995). American preschoolers as young as age 3 years enforce gender norms by intervening peers’ GN play (Langlois and Downs 1980; Xiao et al. 2019). Chinese children aged 4–6 years express a greater interest in becoming friends with GC peers than with GN peers (Qian et al. 2021). Serbian children aged 6–7 years are more likely to demand their peers to end GN behaviors than to accept the behaviors (Skočajić et al. 2020). Children's attitudes toward GN behaviors and GN peers become increasingly negative across childhood (Blakemore 2003; Carter and McCloskey 1984). In America, children aged 4–5 years report lower liking and weaker friendship preferences for GN peers than for GC peers, and these GC–GN differences are even larger in children aged 6–8 years (Riggs et al. 2023). In Hong Kong, children aged 4–5 years rate GN peers as more morally wrong and less popular than GC peers, and these GC–GN differences are even larger in children aged 8–9 years (Kwan et al. 2020).

Children's dislike of GN peers may have its roots in social learning of gender norms. Children internalize societal expectations of “appropriate” gender expressions through observing how parents, peers, and the media reinforce conforming expressions and disapprove gender norm violations (Bussey and Bandura 1999; Giaccardi et al. 2016; Kane 2006; Klemmer et al. 2021; Kowalski 2007; Spivey et al. 2018; Ward and Grower 2020; Xiao et al. 2019). In addition to dislike, children exhibit more hostile behaviors toward GN peers. Although children generally oppose bullying, they may view it as a legitimate punishment or deserved treatment for those who violate social norms (Spence et al. 2024; Teräsahjo and Salmivalli 2003). Children often ostracize and ridicule peers for violating gender norms (Ioverno et al. 2021; Kowalski 2007; Langlois and Downs 1980).

Compared with GC children, children with GN characteristics are at heightened risk for social rejection and bullying victimization (Aspenlieder et al. 2009; Toomey et al. 2014; Zosuls et al. 2016). Experimental studies have reported that children's biases against GN peers are more pervasive in some cultures (e.g., mainland China, Hong Kong) than in other cultures (e.g., Canada, Thailand) (Nabbijohn et al. 2020; Wang et al. 2022). However, across cultures, large‐scale epidemiological and longitudinal research has shown that GN children and adolescents experience increased bullying victimization (Gordon et al. 2018; Klemmer et al. 2021; Lian et al. 2022; Narita et al. 2024; Roberts et al. 2013), which has been associated with increased long‐term mental health difficulties in adolescence and adulthood (Narita et al. 2024; Roberts et al. 2013). These adverse long‐term consequences of victimization based on gender nonconformity warrant investigations into the socio‐cognitive mechanisms underlying children's hostility toward GN peers.

### 1.2 Dehumanization Framework

The dehumanization framework may offer useful insights into the hostility and bullying perpetration against GN individuals. Dehumanization is a socio‐cognitive process of denying or overlooking certain aspects of a person's humanity (Bandura 2002; Haslam and Loughnan 2014) and perceiving a person as falling below the representation of an “ideal” human (Kteily and Landry 2022). There are blatant and subtle forms of dehumanization. People may blatantly dehumanize targets by directly likening them to lower‐status animals (e.g., chimpanzees and insects) (Delbosc et al. 2019; Kteily et al. 2015), objects (e.g., machines) (Bai and Zhao 2024), or even demons (Giner‐Sorolla et al. 2012). Subtle dehumanization refers to an implicit tendency to perceive targets as having fewer human‐typical traits (e.g., agency, warmth, morality, civility) (Haslam 2006; Yang et al. 2015), emotions (e.g., hope, shame) (Leyens et al. 2000), and mental capacities (Epley and Waytz 2010; Waytz et al. 2010). Subtle dehumanization of mental capacities, also known as dehumanized mind perception, involves perceiving targets to experience fewer mental states (e.g., intentions, thoughts, emotions) (Harris and Fiske 2006, 2011).

Dehumanization reduces the moral worth of the dehumanized (Haslam and Loughnan 2014), resulting in a greater endorsement of violence toward the dehumanized (Goff et al. 2008; Kteily et al. 2015; Viki et al. 2013) and a lower willingness to help them when they suffer (Andrighetto et al. 2014; Cuddy et al. 2007). Dehumanization is distinct from prejudice. Targets could be disliked but not dehumanized (Bruneau, Jacoby, et al. 2018; Bruneau, Kteily, et al. 2018) or could be dehumanized but not disliked (Bruneau et al. 2020). There is also a double dissociation in neural underpinnings between dehumanization and prejudice (Bruneau, Jacoby, et al. 2018; Bruneau, Kteily, et al. 2018). Dehumanization independently predicts hostility after controlling for prejudice (Goff et al. 2008; Kteily et al. 2016, 2022). Understanding dehumanization processes is thus crucial for promoting social inclusion of marginalized individuals.

Social psychologists, cognitive scientists, neuroscientists, and philosophers have extensively studied adults’ dehumanization against stigmatized groups, such as immigrants (Kteily et al. 2015; Markowitz and Slovic 2020), Black people (Goff et al. 2008), Muslims (Viki et al. 2013), individuals experiencing homelessness (Harris and Fiske 2006), people with developmental conditions (Kim et al. 2025; Sitruk et al. 2023), and gender/sexual minorities (MacInnis and Hodson 2012; McCarty and Burt 2024). Adults also dehumanize targets who disrupt social order and norms (Fincher and Tetlock 2016; Rodríguez‐Gómez et al. 2022). In particular, in adults, stereotypically male and female targets are perceived as more human‐like than non‐gendered or degendered targets (Martin and Mason 2022, 2023) and possessing more human‐typical traits than gender diverse targets (Tanriverdi et al. 2024) and transgender targets (Gallagher and Bodenhausen 2021). However, there has been limited developmental psychology research examining dehumanization processes in children (McLoughlin 2023).

### 1.3 Prior Studies on Dehumanization in Children

A few studies have examined how children attribute human‐typical traits and emotions to different social groups using conventional rating scales. In Costello and Hodson (2014), Canadian children aged 6–10 years attributed fewer human‐typical traits (e.g., creative, careless) and emotions (e.g., love, guilt) to Black peers than to White peers. In Martin et al. (2008), Scottish children as young as age 6 years expected their own football team to experience more secondary (i.e., human‐typical) emotions than primary emotions (i.e., not human‐typical) but the rival team to experience similar levels of primary and secondary emotions. In Chas et al. (2018), Spanish children aged 10–12 years associated animal‐related words more strongly with Arab names and human‐related words more strongly with Spanish names. In Corbett et al. (2024), Irish children aged 9–11 years reported that autistic peers had fewer human‐typical traits and emotions than non‐autistic peers. Developmental studies based on attribution of human‐typical traits and emotions have been challenged, because the traits and emotions used in these studies were usually derived from studies on adults, presuming that children and adults share a similar understanding of humanness and the chosen traits and emotions (McLoughlin 2023).

In recent years, developmental studies have started to examine children's dehumanization using visual scales and behavioral measures that do not necessitate an advanced understanding of human‐typical traits and emotions. This line of research has revealed that young children, like adults, are capable of dehumanizing others both blatantly and subtly. In Zhou and Hare (2022), American children aged 5–12 years were more likely to associate non‐human illustrations with outgroup peers depicted as incompetent and immoral than with ingroup peers on visual scales. Also, children who blatantly dehumanized the outgroup members recommended more severe punishments for their misbehaviors (Zhou and Hare 2022). In McLoughlin et al. (2018), based on a British sample, 6‐year‐olds, but not 5‐year‐olds, rated the faces of a gender or geological outgroup as having less humanness than those of an ingroup. In McLoughlin and Over (2017), when describing a gender or geological outgroup compared with an ingroup, British children aged 5 and 6 years produced fewer mental state words and those aged 6 years also produced less diverse mental state words. In Gönültaş et al. (2020), Turkish children aged 9–13 years were better at mentalizing the minds of culturally dissimilar and non‐stigmatized (i.e., Northern European) group members than culturally similar and stigmatized (i.e., Syrian) group members, suggesting that dehumanization may not be entirely dependent on similarity or ingroup/outgroup differentiation but also other characteristics such as social status and perceived threat of the targets. Given that most prior studies on children focused on relatively straightforward ingroup/outgroup differentiation, further research is needed to examine the relative importance of group membership and other target characteristics, such as social norm violation, in driving dehumanization processes.

It is noteworthy that prior studies have not yielded consistent findings on developmental trends of dehumanization processes. Some studies did not find any age effects on dehumanization (van Noorden et al. 2014; Zhou and Hare 2022). Some studies reported that dehumanization may emerge at age 5 or 6 years, depending on the outcome measure employed (McLoughlin and Over 2017; McLoughlin et al. 2018). Further research on the developmental trajectory of dehumanization is needed.

### 1.4 Present Study

No research to date has investigated children's dehumanization of GN peers and its developmental trajectory. Also, most prior studies on children employed measures of either blatant or subtle dehumanization but did not assess both forms of dehumanization simultaneously. It has been proposed that blatant and subtle forms of dehumanization may reflect distinct mechanisms in both children and adults and may be associated with different cognitive processes and downstream behavioral outcomes (Bruneau et al. 2020; Kteily et al. 2015; Zhou et al. 2024). A dual assessment of both blatant and subtle dehumanization can facilitate the comparison of their developmental trajectories and their relative importance in predicting bullying tendency. A dual assessment may also inform future development of interventions, offering insights into target mechanisms.

The present study examined whether children in early to late childhood attribute humanness differently based on peer gender and peer gender conformity using measures of both blatant and subtle dehumanization. This paradigm based on both peer gender and peer gender conformity can shed light on the contributions of ingroup/outgroup differentiation (peer gender) as well as social norm violation (gender nonconformity) to children's dehumanization. Also, to the authors’ best knowledge, all existing published studies on dehumanization in children have been conducted in Western and/or European cultures. Although the present study was not designed to test any cross‐cultural perspectives, the present study was among the first to contribute relevant evidence on children's dehumanization from an East Asian setting.

In the present study, children from four age groups (5–6, 7–8, 9–10, and 11–12 years) in Hong Kong were recruited to learn about vignettes of four hypothetical peers who varied in gender (boy, girl) and gender conformity (GC, GN). The youngest age group was set at 5–6 years, because this is the youngest age at which previous studies found children to dehumanize others (McLoughlin and Over 2017; Zhou and Hare 2022). The present study included children aged 5–12 years to explore developmental patterns from early to late childhood. This age range is identical to the age range examined in a relatively comprehensive prior study on dehumanization in children (Zhou and Hare 2022).

After learning the peer vignettes, children completed dehumanization tasks and answered questions about bullying tendency based on the peers. The insect scale (Delbosc et al. 2019) was employed to assess blatant dehumanization. The mind perception task (McLoughlin and Over 2017) was adopted to measure subtle dehumanization. While subtle dehumanization may involve the denial of human‐typical traits and emotions, the present study focused on mind perception, because mind perception assessment for children does not necessarily require children to possess adult‐like conceptions of human‐typical traits and emotions or an understanding of sophisticated trait and emotion words.

#### 1.4.1 Prediction and Exploration

##### 1.4.1.1 Peer Gender Conformity

As reviewed previously, children tend to exclude and bully peers who violate gender norms. Given that dehumanization is associated with bullying in children (Pozzoli et al. 2012; Thornberg and Jungert 2014; van Noorden et al. 2014) and that dehumanization can be activated by norm violations (Fincher and Tetlock 2016), it is possible that children dehumanize peers who violate gender norms. Based on these prior findings, it was hypothesized that children would rate GN peers as less human‐like/more insect‐like than GC peers on the insect scale (i.e., blatant dehumanization) and would spontaneously attribute fewer and less diverse mental states to GN peers than to GC peers in the mind perception task (i.e., subtle dehumanization).

##### 1.4.1.2 Age Group

Given that prior developmental studies yielded inconsistent findings on age effects and did not examine GN‐based dehumanization, there were no specific hypotheses concerning age group. Instead, the present study was designed to explore and document the related developmental patterns across age groups.

##### 1.4.1.3 Peer Gender

An exploratory approach was adopted to examine the effects of peer gender. Some researchers propose that dehumanization arises primarily from ingroup/outgroup differentiation and is typically directed toward outgroups (Leyens et al. 2000; McLoughlin et al. 2018; Zhou and Hare 2022). Other researchers propose that not all outgroups are dehumanized and that some target characteristics may interact with group membership to influence humanness attribution (Bruneau, Jacoby, et al. 2018; Bruneau, Kteily, et al. 2018; Gönültaş et al. 2020; Morehouse et al. 2023).

###### 1.4.1.3.1 Peer Gender: Potential Direction 1

If dehumanization is primarily driven by simple ingroup/outgroup differentiation, one would expect to detect children's dehumanization of other‐gender peers. The present study examined both peer gender and peer gender conformity. If peer gender and peer gender conformity are similarly strong forces in driving dehumanization, one would expect to detect children's dehumanization of other‐gender peers as well as GN peers. In this context, one might also expect to detect stronger dehumanization of other‐gender GN peers (additive effects of double outgroups) than own‐gender GN peers (a single outgroup).

###### 1.4.1.3.2 Peer Gender: Potential Direction 2

Alternatively, compared with simple ingroup/outgroup differentiation, social norm violation may be a much stronger driving force. In prior research, it has been found that the influences of peer gender conformity can override those of other social categories on children's peer evaluations and friendship preferences (Qian et al. 2021), which is in line with the observation that gender norm violations are highly salient to children (Blakemore 2003). If peer gender conformity overrides peer gender in driving dehumanization, one would expect to detect children's dehumanization of GN peers (both own‐gender and other‐gender) but not all other‐gender peers. In other words, in this scenario, children would dehumanize GN peers regardless of peer gender.

##### 1.4.1.4 Dehumanization and Bullying

Prior studies have shown that children's dehumanization is associated with increased hostility toward the dehumanized targets (van Noorden et al. 2014; Zhou and Hare 2022). The present study therefore also examined whether children's dehumanization could explain their propensity to bully GN peers. Dehumanization may enable harmful acts by making inhumane treatment against targets more justified and reducing perceivers’ moral distress that might otherwise restrain violent behaviors (Bandura 2002; Haslam and Loughnan 2014). It was hypothesized that dehumanization would mediate the relationship between peer gender conformity and bullying tendency. In order to establish the direction of mediation effects, reverse mediation models with bullying tendency as the mediator and dehumanization as the outcome were also examined to compare the different models (dehumanization as mediator vs. bullying as mediator).

## 2 Methods

### 2.1 Participants

Participants were 472 ethnically Chinese children in Hong Kong across four age groups: 5–6 years (*M* = 5.53, *SD* = 0.50), 7–8 years (*M* = 7.52, *SD* = 0.50), 9–10 years (*M* = 9.34, *SD* = 0.48), and 11–12 years (*M* = 11.19, *SD* = 0.39). Participants in each age group consisted of 59 girls and 59 boys. According to G*Power 3.1 (Faul et al. 2009), based on the current study design, a sample of 472 participants is required to detect a small effect size (*f* = 0.1) with 90% power (*α =* 0.05, *r* = 0.30, nonsphericity correction *ε* = 1). Data collection was terminated immediately when the required sample size was reached.

Participants were recruited through a university, local schools, community centers, and online. All participants were residing in Hong Kong, an urban community that is ethnically predominantly Chinese. It was estimated that the median monthly domestic household income in Hong Kong in 2021 was HKD 27,320 (approx. USD 3515) (Census and Statistics Department 2023). Participant‐level socioeconomic data were not measured. However, based on profiles of the schools/community centers involved and prior studies conducted by the authors using similar recruitment procedures, it is likely that most participants came from households with average or above‐average income.

### 2.2 Procedures

The study was approved by the Human Research Ethics Committee at the University of Hong Kong. Informed parental consent and child assent were obtained. All participants were tested in person individually in a quiet room in their school, a community center, or at the researchers’ university.

**FIGURE 1:**
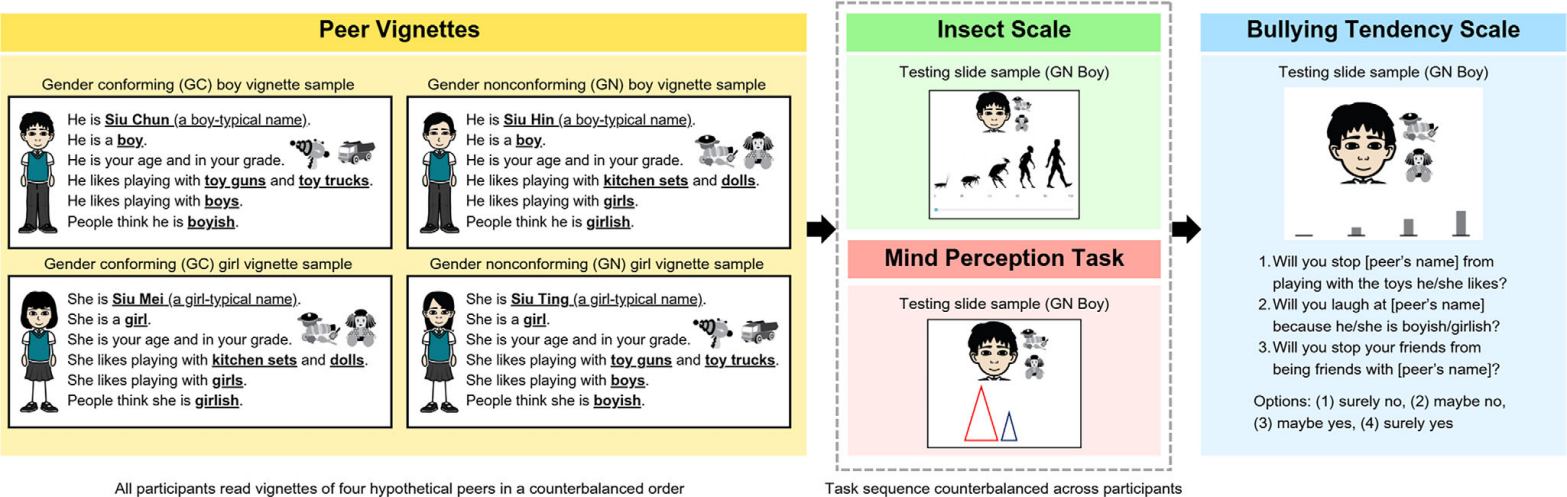
Schematic diagram of the experimental design and sample slides.

After warming up, each participant completed four tasks: starting with peer vignettes, then insect scale and mind perception task, and finally bullying tendency scale (see Figure 1). The order of the peer vignettes, as well as the order of the insect scale and mind perception task, was counterbalanced across participants. After completing each task, participants received a stamp on a token economy chart. Participants received a small gift (e.g., stationery, stickers) and a participation certificate after completing the experiment. The entire testing session lasted around 30–40 min.

#### 2.2.1 Peer Vignettes Manipulation

Each participant was shown four peer vignettes (GC boy, GN boy, GC girl, GN girl) on laminated cards one by one, in an order counterbalanced across participants. The written descriptions of the four peer vignettes were read aloud by the experimenter. The peer vignettes consisted of background information of two boy avatars and two girl avatars who were either GC or GN. The avatars were created using the Storyboard That online website. Each boy or girl avatar was GC for half of the participants and GN for the other half (i.e., the pairing of different avatars and GC/GN characteristics was also counterbalanced). The gender of each peer was explicitly stated in the written descriptions and further indicated by their names, hairstyles, school uniforms, and gender pronouns. Peer gender conformity was manipulated by the peers’ gender‐typed toy preferences and playmate preferences, which show large differences between boys and girls according to prior meta‐analytic research and large‐scale population studies (Davis and Hines 2020; Golombok et al. 2008; Iervolino et al. 2005; Kung et al. 2024). The GC boy and the GN girl shared identical boy‐typical preferences (i.e., toy gun, toy truck, playing with boys), while the GC girl and the GN boy shared identical girl‐typical preferences (i.e., doll, kitchen set, playing with girls). Grayscale toy icons were included in the vignettes to facilitate children's understanding of the gender conformity of those hypothetical peers. All the target peers were depicted as Asian/Chinese‐looking children of the same age and in the same grade as the participant. For example, this is the description of the peer vignette for the GN boy:

“He is Siu Hin (a boy‐typical name). He is a boy. He is your age and in your grade. He likes playing with kitchen sets and dolls. He likes playing with girls. People think he is girlish.”

After seeing each vignette, participants were asked three attention‐check questions regarding the peer's gender‐related preferences and expressions: (1) “Does [peer's name] like playing with toy guns and toy trucks or kitchen sets and dolls?” (2) “Does [peer's name] like playing with boys or girls?” (3) “Do people think [peer's name] is boyish or girlish?” If a participant answered any question incorrectly, the same peer vignette would be shown and read again, and then the participant would be asked the questions again. Participants who failed the attention‐check twice for any hypothetical peer were excluded from analyses.

#### 2.2.2 Insect Scale

The insect scale (Delbosc et al. 2019) was adopted to measure perceived blatant humanness of the peers. Compared with the more well‐known ape scale (Kteily et al. 2015), the insect scale may provide a stronger test of blatant dehumanization, as children show lower concern for the lives of invertebrates compared to primates and humans (Henseler Kozachenko and Piazza 2021; McGuire et al. 2023). The insect scale contains a continuous slider ranging from 0 (insect) to 100 (human), with five silhouettes depicting a gradation of humanness from an insect to a full human being (see Figure 1). Lower scores on the insect scale indicate greater levels of blatant dehumanization. The insect scale was presented using the Qualtrics platform. The experimenter introduced the scale on the computer screen by saying:

“We are all humans, but some of us might think some people are more like cockroaches, and others are more like humans. We can use this scale to tell how much we think a person is like a cockroach or a human. A ‘0’ (move the slider to 0) means you think someone is very much like a cockroach. A ‘100’ (move the slider to 100) means you think someone is very much like a human. A lower score means someone is more like a cockroach. A higher score means someone is more like a human.”

In a practice exercise, all participants were asked to try to move the slider and to identify the endpoints of the scale. All participants were able to move/use the slider properly and accurately identify the corresponding endpoints of “very much like a cockroach” and “very much like a human.” Terms such as “insects” or “bugs” were not used in the instructions, because these terms may be overly broad, and “insects” is not a commonly used word in everyday life. “Cockroach” was chosen, because it is not uncommon to see cockroaches in Hong Kong due to its hot and moist weather, and cockroach is something local children can easily understand.

Participants then completed the insect scale for all hypothetical peers (one peer on each page), in an order identical to the presentation sequence of peer vignettes. When rating a peer, participants were presented with a slide consisting of the insect scale and an icon of the peer above the scale. Icons of the peer's preferred toys were shown beside the peer icon as a visual cue to remind participants of the peer's gender (non)conformity. The experimenter said, “*He/she is [peer's name]. He/she is a boy/girl who is boyish/girlish. From 0 to 100, how much do you think [peer's name] is like a human?*” The experimenter told participants there was no right or wrong answer and their answers could be the same or different across hypothetical peers. The experimenter would look away from the screen when the participant rated a peer to minimize social desirability biases.

#### 2.2.3 Mind Perception Task

Mental state attribution to hypothetical peers was assessed using the mind perception task, which is a child‐friendly task and has been used to elicit mental state words in young children (McLoughlin and Over 2017). The mind perception task involves four standardized videos, each presenting a unique set of pre‐programmed sequences of actions. The interesting and dynamical actions presented in these videos are visual prompts designed to elicit mentalization (Abell et al. 2000). Notably, some prior research measured participants’ mental state attribution by asking them to imagine a typical day of the target (Harris and Fiske 2011). However, given the current repeated measures design of the peer conditions, it would be relatively challenging for children to imagine a typical day of the target repeatedly for four hypothetical peers without any visual prompts and to repeatedly produce meaningful verbal responses for analyses. Thus, in the present study, the relatively child‐friendly mind perception task was employed.

Frith–Happé animations (Abell et al. 2000), approximately 40‐s videos depicting ambiguous interactions between one big triangle and one small triangle, were used in this task. These videos could be described using simple action terms (e.g., they ran together) or mental state words (e.g., they were happy). Subtle dehumanization occurs when targets associated with the triangles are described with significantly fewer and less diverse mental state words than other targets.

In a practice trial, participants were first shown a slide with one bigger triangle and one smaller triangle. The experimenter told the participants, *“This video is about two children. This is a child (pointing at the bigger triangle). This is another child (pointing at the smaller triangle)*.” Then, the experimenter played a Frith–Happé animation designed for practice once. Then, the experimenter showed the slide with two triangles again and asked participants the following questions one by one to elicit their descriptions of the activities of the triangles:

“What do you think was happening in the video?”

“What do you think the children were doing?”

“Tell me about this child (pointing at the bigger triangle); how was the child?”

“Tell me about this child (pointing at the smaller triangle); how was the child?”

After practice, participants watched four Frith–Happé animations one by one, each associated with one of the peers introduced. The presentation order is identical to that in the peer vignette task. The pairing of animations and peers was counterbalanced across participants. For each peer, participants were first shown a slide with an icon of the peer, icons of the peer's preferred toys, and two triangles (see Figure 1). For example, for the GN girl, the experimenter introduced the video by saying:

“This video is about Siu Ting (girl‐typical name) and another child like her. This is Siu Ting (pointing at the bigger triangle). This is the other child (pointing at the smaller triangle). They are both girls who are boyish (pointing at the toy icons and avatars).”

In other words, the two triangles in each video represented peers of the same gender with the same gender conformity characteristics. In each trial, after watching the video, participants were shown the same slide with the peer icons, toy icons, and triangles, which were shown to them at the beginning of the trial, to remind them of whom the triangles represented in that video. Then, participants were asked the same questions used in the practice trial, except that the identity of the bigger triangle was specified (e.g., “*Tell me about Siu Ting (pointing at the bigger triangle); how was the child?*”). Participants’ responses were audio‐recorded for coding of mental state words. They were encouraged to speak freely and assured that the recordings would not be shared with their family or teachers.

#### 2.2.4 Bullying Tendency Scale

Based on previous research on children's behavioral maltreatment against GN peers (Carter and McCloskey 1984; Langlois and Downs 1980), a 3‐item measure was developed to measure participants’ tendency to bully each peer based on their gender (non)conformity. These items include: (1) “Will you stop [peer's name] from playing with the toys he/she likes?” (2) “Will you laugh at [peer's name] because he/she is boyish/girlish?” and (3) “Will you stop your friends from being friends with [peer's name]?” The bullying tendency scale was presented using the Qualtrics platform. When rating a peer, icons of the peer and his/her preferred toys were shown near the top of the screen. The experimenter reminded participants of the gender and gender conformity of the target peer and then read aloud each question. Participants then selected an option from “surely no,” “maybe no,” “maybe yes,” and “surely yes.” Four bars of different lengths were shown above the options to help participants understand the gradation of different options (see Figure 1). The experimenter reminded participants that there were no correct or wrong answers. A bullying tendency score was computed by averaging the responses to the questions, with higher scores indicating greater bullying tendency. The scale had good internal consistency (*α* = 0.71).

### 2.3 Data Handling and Analysis Plan

#### 2.3.1 Coding of Mental State Words

A coding scheme of mental state words (see Supporting Information) was developed based on previous research that measured the mental state content of speech (Abell et al. 2000; Chan et al. 2020; McLoughlin and Over 2017; Meins et al. 2013). Mental state words included words describing desires and intentions (e.g., love/like, want), emotions (e.g., happy, grateful, sad, regret), cognitions (e.g., think, realize), actions that involve interactions of mental states (e.g., comfort, tell lies), and personality traits (e.g., friendly, selfish).

After coding, frequency proportions of mental state words were calculated by dividing the number of mental state words by the total number of words produced. Diversity proportions of mental state words were calculated by dividing the number of non‐repeated mental state words by the total number of words produced. Both frequency and diversity of mental state words were examined, because the quantity and variety of mental state words may exhibit distinct developmental patterns (McLoughlin and Over 2017). The proportion measures were chosen over the absolute frequency measures as proportional scores could take into account the length of each response.

To train coders, the first author created a fictitious transcript and then demonstrated how to identify mental state words using the coding scheme, as well as how to record the frequency and diversity of these words on a computer sheet. The coders were then asked to practice these steps using another fictitious transcript and engaged in discussions to resolve any inconsistencies. After training, the first author coded all the transcripts based on the coding scheme, and the other three coders independently coded 10% of randomly selected transcripts (i.e., 10% of the transcripts were independently coded by four people). All coders were blinded to the conditions (i.e., participant gender, age group, peer gender, and peer gender conformity) of the transcripts while coding. Inter‐coder reliability across the four coders was assessed by single‐measure intraclass correlations (ICCs) using two‐way mixed effect models with absolute agreement. The inter‐coder reliability was high for both frequency proportion (ICC = 0.85, 95% CI [0.79, 0.89], *p* < 0.001) and diversity proportion measures (ICC = 0.90, 95% CI [0.86, 0.93], *p* < 0.001).

### 2.4 Statistical Analyses

Nine participants were excluded from all analyses because they failed the attention‐check questions of at least one peer vignette twice (*n* = 7) and were inattentive (*n* = 2). Six participants were excluded from the analyses of the mind perception task due to speech problems (*n* = 2) and technical errors (*n* = 4). Following statistical guidelines (Tabachnick and Fidell 2019) and recent research studies in the area of developmental science (e.g., Bernier et al. 2024; Clinchard et al. 2024; Meijer et al. 2021), data points with *z* ≥ 3.29 or ≤−3.29 on each measure were treated as outliers and excluded from the respective analyses (blatant humanness rating: *n* = 19; frequency proportion: *n* = 17; diversity proportion: *n* = 22; bullying tendency: *n* = 16). Analyses based on winsorized data (i.e., outliers were included and converted to the nearest non‐outlier values) yielded highly similar results (see Supporting Information for findings based on winsorized data).

All statistical analyses were run in SPSS version 29.0.2.0. The distributions of blatant humanness ratings for all hypothetical peer conditions were left‐skewed, and those for the frequency proportion, diversity proportion, and bullying tendency were right‐skewed. Following the analytical approach employed in prior developmental research on skewed data (Brandone and Wellman 2009; Tolmatcheff et al. 2022), we first conducted parametric analyses and then confirmed the significant results using nonparametric analyses (see Supporting Information for procedures and results of nonparametric analyses).

For parametric analyses, a series of 4 (Age Group: 5–6, 7–8, 9–10, and 11–12 years; between‐subject) × 2 (Participant Gender: boy vs. girl; between‐subject) × 2 (Peer Gender: boy vs. girl; within‐subject) × 2 (Peer Gender Conformity: gender conforming vs. gender nonconforming; within‐subject) mixed ANOVAs were conducted for each dependent variable (i.e., blatant humanness rating, frequency proportion, diversity proportion, bullying tendency). Significant interactions were examined with planned post hoc pairwise comparisons. Effect sizes of pairwise comparisons were estimated using Cohen's *d* generated by separate independent or paired sample *t*‐tests, with cutoffs of 0.2, 0.5, and 0.8 indicating small, medium, and large effects, respectively (Cohen 1988).

Mediation analyses, including multilevel moderated mediation analyses, were conducted using MLmed SPSS macro (Rockwood and Hayes 2017) to examine whether the humanness measures mediated the relationship between peer gender conformity and bullying tendency. Potential moderators (e.g., age group) would be identified in the mixed ANOVAs. For instance, if children's dehumanization of GN peers varied across age groups, age group would be included as the moderator in multilevel moderated mediation models. Multilevel models were tested separately for each humanness mediator (i.e., blatant humanness rating, frequency proportion of mental state words, diversity proportion of mental state words). Monte‐Carlo simulations generating Restricted Maximum Likelihood 95% confidence intervals (CIs) using 10,000 resamples were conducted to estimate the model effects. If the index of moderated mediation was significant, mediation models were tested separately for each level/group of the moderator to examine the differences in indirect/mediating effects across levels/groups of the moderator. Reverse mediation models were also tested (see Supporting Information).

## 3 Results

The parametric and nonparametric analyses yielded highly similar results. For ease of interpretation, only results from the parametric analyses are presented below. Nonparametric analyses are reported in Supporting Information.

### 3.1 Blatant Humanness Ratings

**FIGURE 2:**
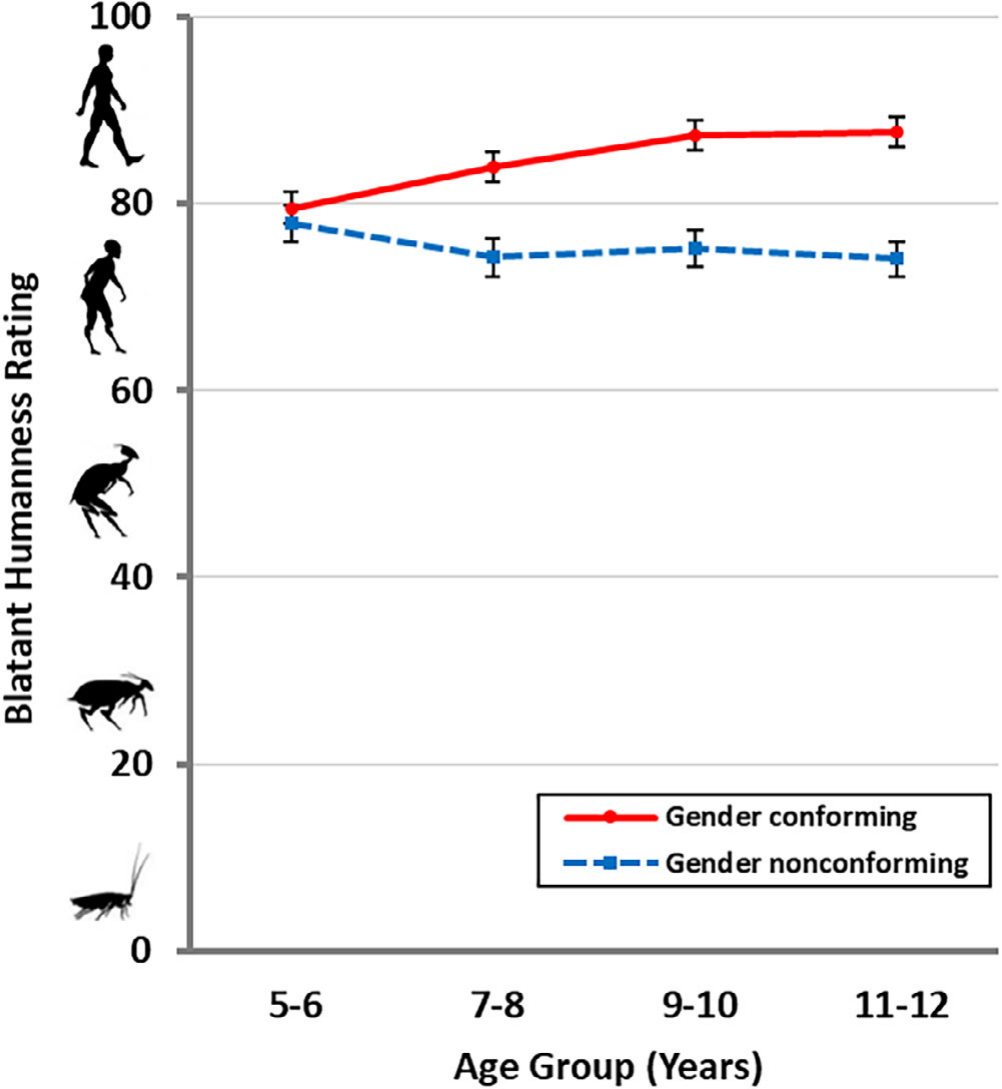
Mean blatant humanness ratings as a function of age group and peer gender conformity. Error bars represent standard error.

There was a significant main effect of peer gender conformity, *F*(1, 436) = 79.86, *p* < 0.001, η_p_^2^ = 0.15, showing that participants rated GN peers (*M* = 75.3, *SD* = 20.7) as less human‐like/more insect‐like than GC peers (*M* = 84.6, *SD* = 17.2). There was also a significant interaction between peer gender conformity and age group, *F*(3, 436) = 6.45, *p* < 0.001, η_p_^2^ = 0.04 (see Figure 2). Post hoc pairwise comparisons suggest that significant blatant humanness rating differences between GC and GN peers were present in older but not younger children. Participants in the 5–6 years age group did not rate GC and GN peers differently (*p* = 0.456, *d* = −0.07). However, participants in the older age groups rated GN peers as less human‐like/more insect‐like than GC peers (7–8 years: *p* < 0.001, *d* = −0.48; 9–10 years: *p* < 0.001, *d* = −0.71; 11–12 years: *p* < 0.001, *d* = −0.74). All other main effects and interactions were non‐significant (see Supporting Information for detailed findings on other main effects and interactions).

### 3.2 Mental State Word Proportions

The main effect of age group was significant for both frequency proportion, *F*(3, 432) = 17.64, *p* < 0.001, *η_p_* ^2^ = 0.11, and diversity proportion measures, *F*(3, 427) = 16.01, *p* < 0.001, *η_p_* ^2^ = 0.10, such that older children in general produced a higher proportion of and more diverse mental state words than did younger children (see Supporting Information for detailed findings on age group comparisons).

**FIGURE 3:**
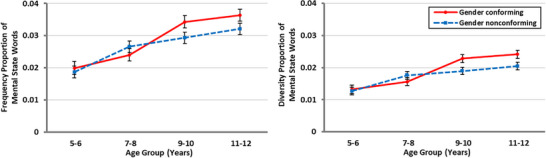
Mean frequency proportion (left) and diversity proportion (right) of mental state words as a function of age group and peer gender conformity. Error bars represent standard error.

There were significant main effects of peer gender conformity on frequency proportion, *F*(1, 432) = 4.02, *p* = 0.046, *η_p_* ^2^ = 0.01, and on diversity proportion, *F*(1, 427) = 6.15, *p* = 0.014, *η_p_* ^2^ = 0.01. Participants used fewer mental state words to describe GN peers (*M* = 0.027, *SD* = 0.019) than GC peers (*M* = 0.029, *SD* = 0.020) and used less diverse mental state words to describe GN peers (*M* = 0.017, *SD* = 0.012) than GC peers (*M* = 0.019, *SD* = 0.013). In addition, the peer gender conformity × age group interactions were significant for both the frequency proportion measure, *F*(3, 432) = 3.10, *p* = 0.027, *η_p_* ^2^ = 0.02, and diversity proportion measure, *F*(3, 427) = 4.90, *p* = 0.002, *η_p_* ^2^ = 0.03 (see Figure 3). Post hoc pairwise comparisons revealed that participants in the younger age groups (5–6 years, 7–8 years) used similar proportions of mental state words to describe GC and GN peers (5–6 years: *p* = 0.552, *d* = −0.06; 7–8 years: *p* = 0.191, *d* = 0.13), and similarly diverse mental state words to describe GC and GN peers (5–6 years: *p* = 0.659, *d* = −0.04; 7–8 years: *p* = 0.129, *d* = 0.14). However, children in the older age groups (9–10 and 11–12 years) used fewer mental state words to describe GN peers than to describe GC peers (9–10 years: *p* = 0.011, *d* = −0.26; 11–12 years: *p* = 0.028, *d* = −0.23) and used less diverse mental state words to describe GN peers than to describe GC peers (9–10 years: *p* = 0.002, *d* = −0.32; 11–12 years: *p* = 0.003; *d* = −0.30). All other main effects and interactions were non‐significant (see Supporting Information for detailed findings on other main effects and interactions).

### 3.3 Bullying Tendency

**FIGURE 4:**
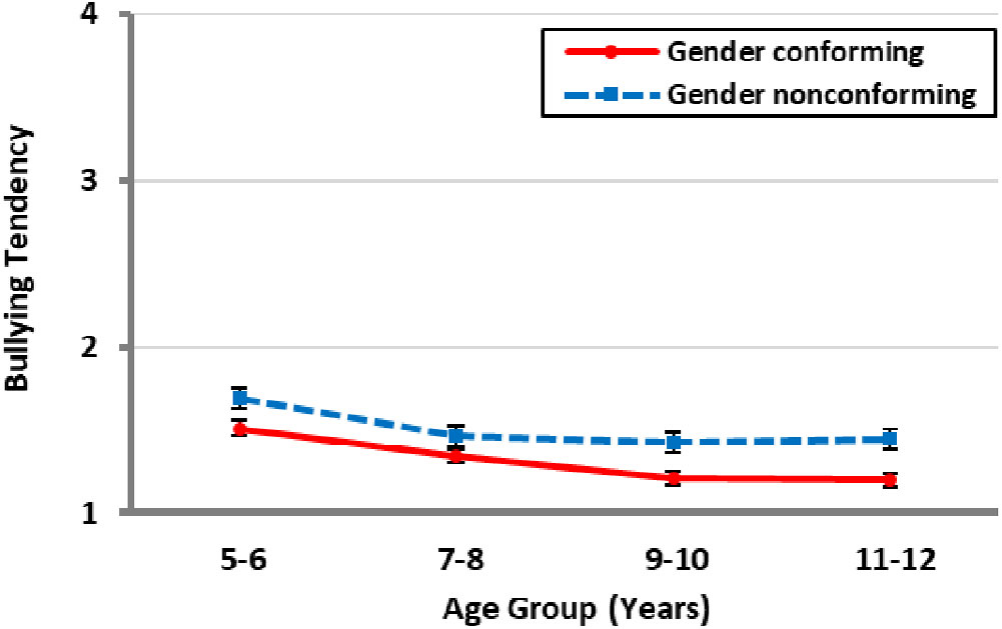
Mean bullying tendency as a function of age group and peer gender **conformity**. Error bars represent standard error.

The main effect of age group was significant, *F*(3, 441) = 8.03, *p* < 0.001, *η_p_* ^2^ = 0.05. Post hoc analyses showed that participants aged 5–6 years reported higher bullying tendency than the other age groups (all *p* < 0.01; see Supporting Information). The main effect of peer gender conformity was also significant, *F*(1, 441) = 60.17, *p* < 0.001, *η_p_* ^2^ = 0.12 (see Figure 4). Participants reported a stronger tendency to bully GN peers (*M* = 1.5, *SD* = 0.7) than GC peers (*M* = 1.3, *SD* = 0.4). The age group × participant gender interaction was significant, *F*(3, 441) = 2.91, *p* = 0.034, *η_p_* ^2^ = 0.02. Boy participants reported a stronger bullying tendency than girl participants in the 5–6 years age group (*p* < 0.001), but not in the other age groups (see Supporting Information). The peer gender × peer gender conformity × participant gender interaction was also significant, *F*(1, 441) = 6.65, *p* = 0.010, *η_p_* ^2^ = 0.02. To investigate this interaction, separate peer gender × participant gender ANOVAs were conducted for GC and GN peer conditions. For GC peers, the peer gender × participant gender interaction was significant, *F*(1, 447) = 9.27, *p* = 0.002, *η_p_* ^2^ = 0.02; but for GN peers, the peer gender × participant gender interaction was non‐significant, *F*(1, 447) = 0.50, *p* = 0.481, *η_p_* ^2^ = 0.00. While boy participants reported weaker bullying tendency toward GC boys than GC girls (*p* = 0.002), girl participants showed no differences in bullying tendency for GC boys and girls (*p* = 0.265). Neither boy nor girl participants showed different bullying tendency for GN boys and girls (see Supporting Information).

### 3.4 Multilevel Moderated Mediation

**FIGURE 5:**
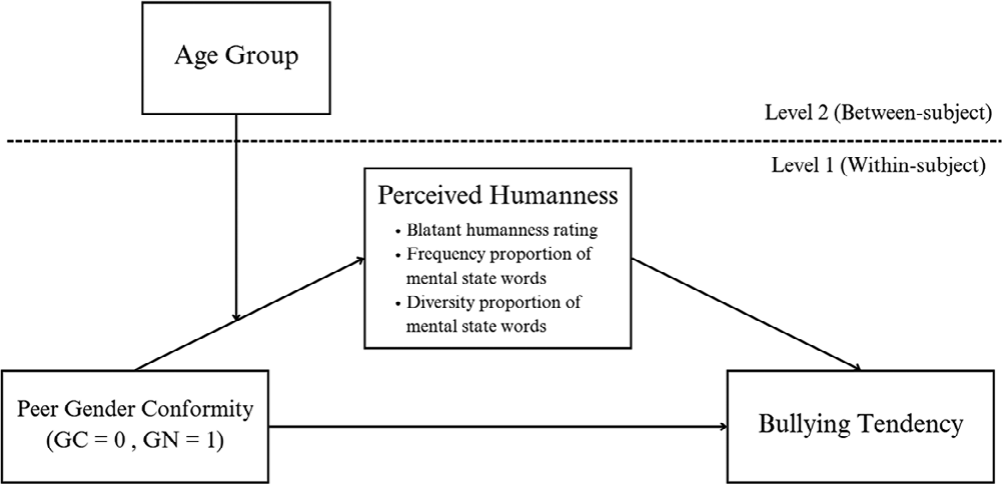
Multilevel moderated mediation model.

Multilevel moderated mediation models were run to examine whether peer gender conformity predicted bullying tendency via perceived humanness (i.e., blatant humanness rating, frequency proportion, and diversity proportion) and whether the age group of participants moderated this mediation. For each measure, scores for GC boys and girls were averaged to form a single GC score variable, and those for GN boys and girls were averaged to form a single GN score variable. Scores for boy peers and girl peers were combined because there were no significant main effects or interactions involving peer gender for either blatant or subtle dehumanization. The predictor (i.e., peer gender conformity: GC = 0; GN = 1), humanness mediators, and the outcome variable (i.e., bullying tendency) were at level 1, nested within each participant; the age group of participants (coded as an ordinal variable of 1–4) was a level 2 variable proposed to moderate the relationship between the predictor and mediators. Between‐subject effects were omitted from all analyses because there were no interindividual differences in the predictor; all participants rated both GC and GN peers. The conceptual model is depicted in Figure 5.

The index of moderated mediation was significant when blatant humanness rating (estimate = 0.021, 95% CI [0.009, 0.035]) was the mediator of the model. The index of moderated mediation was non‐significant when frequency proportion (estimate = −0.004, 95% CI [−0.011, 0.001]) or diversity proportion (estimate = −0.003, 95% CI [−0.011, 0.003]) was included as the mediator. Each mediation model was also tested at different age group levels. Results revealed that the indirect effects through blatant humanness rating were significant in the 9–10 and 11–12 years age groups, but not in the 5–6 and 7–8 years age groups (see Table 1). Indirect effects via frequency and diversity proportion were non‐significant across all age groups (see Table 1). Reverse mediation models using bullying tendency as a mediator and blatant humanness rating as an outcome yielded less significant indirect effects in the 9–10 and 11–12 years age groups (see Supporting Information), and thus the original model with blatant humanness rating as a mediator was retained.

**TABLE 1:** Indirect effects of the mediation models by age group.

				Indirect effects				
Predictor	Outcome	Mediator	Age group	*b*	*SE*	*Z*	*p*	95% CI
Gender conformity (GC = 0, GN = 1)	Bullying tendency	Blatant humanness rating	5–6 years	0.003	0.009	0.296	0.767	−0.015, 0.025
			7–8 years	0.033	0.021	1.541	0.123	−0.005, 0.081
			9–10 years	0.061	0.024	2.504	**0.012**	**0.017, 0.114**
			11–12 years	0.108	0.033	3.304	**0.001**	**0.048, 0.180**
		Frequency proportion of mental state words	5–6 years	−0.006	0.017	−0.342	0.733	−0.043, 0.027
			7–8 years	0.006	0.010	0.614	0.539	−0.009, 0.029
			9–10 years	−0.008	0.011	−0.723	0.470	−0.033, 0.012
			11–12 years	0.011	0.012	0.905	0.365	−0.010, 0.040
		Diversity proportion of mental state words	5–6 years	−0.002	0.016	−0.138	0.890	−0.035, 0.028
			7–8 years	0.003	0.010	0.299	0.765	−0.016, 0.026
			9–10 years	−0.004	0.013	−0.305	0.760	−0.031, 0.022
			11–12 years	0.016	0.017	0.987	0.324	−0.014, 0.054

Abbreviations: *b*, unstandardized regression coefficient; CI, confidence interval; GC, gender conforming; GN, gender nonconforming; *SE*, standard error.

Following a peer review suggestion, a multilevel parallel moderated mediation model including all three humanness measures (i.e., blatant humanness rating, frequency proportion, diversity proportion) as simultaneous mediators was also tested to compare the indirect effects of different humanness measures (see Supporting Information). Furthermore, as there was a significant peer gender × peer gender conformity × participant gender interaction in predicting bullying tendency, an additional model including participant gender and peer gender as moderators of the path between gender conformity and bullying tendency of the original model (see Figure 5 for the original model) was also tested (see Supporting Information). Consistent with the original model, only indirect effects via blatant humanness rating in 9–10 and 11–12 years age groups were significant. Thus, for ease of interpretation, the more parsimonious original model (i.e., Figure 5) was retained.

## 4 Discussion

### 4.1 Summary of Findings

The present study was the first to examine children's dehumanization of GN peers. It was found that, by age 9–10 years, children both blatantly and subtly dehumanized GN peers. Older children blatantly rated GN peers as less human‐like/more insect‐like than GC peers. Older children also used fewer and less diverse mental state words to describe videos associated with GN peers than those associated with GC peers. It is noteworthy that there were no main effects or interactions involving peer gender for dehumanization outcomes, suggesting that older children's dehumanization was not dependent on peer gender. More specifically, older children dehumanized GN peers, regardless of peer gender. These findings support the hypothesis that children dehumanize GN peers. These findings also unveil the developmental trajectories of the related dehumanization processes. In older children, blatant humanness ratings, but not mental state word proportions, partially mediated the relationship between peer gender conformity and bullying tendency. These findings provide partial support for the hypothesis that dehumanization plays a mediating role in the relationship between peer gender conformity and bullying.

### 4.2 Potential Explanations and Links to Broader Literature

#### 4.2.1 Developmental Shift in Dehumanization: Younger Versus Older

In the present study, older but not younger children dehumanize GN peers. This developmental shift may be explained by prior observations reported in the broader literature on gender development. At first glance, this pattern seems inconsistent with certain well‐known findings in the field of gender development, such as the increase in gender stereotype flexibility from middle to late childhood (Conry‐Murray and Turiel 2012; Trautner et al. 2005). However, it is noteworthy that, despite increasing cognitive flexibility regarding gender norms, older children continue to see overrepresentation of gender stereotypical expressions in the media (Giaccardi et al. 2016; Ward and Grower 2020), witness adults and peers condemn GN behaviors (Kane 2006; Klemmer et al. 2021), and experience pressure to conform to traditional gender norms (Schroeder and Liben 2021). There is also increasing gender segregation across childhood, which may amplify older children's pressure to conform to gender norms (Masters et al. 2021; Schroeder and Liben 2021; Sroufe et al. 1993) and enhance their scrutiny of peer gender norm violations (Kowalski 2007). These cumulative social pressures, policing, representation, and segregation experienced by older children may contribute to their disapproval and dehumanization of GN peers.

In addition, children, especially older children, appraise GC peers as having higher status, moral standing, competence, and sociability compared with GN peers (Kwan et al. 2024, 2020; Riggs et al. 2023). These qualities are commonly associated with the human ideal (Chu and Martin 2021; Formanowicz et al. 2018; Kteily and Landry 2022). The association between these human‐ideal qualities and gender conformity may also contribute to older children's dehumanization of GN peers.

Taken together, the current findings suggest that, as children grow, they increasingly perceive gender conformity as a sign of humanness and gender nonconformity as deviance from ideal humanness. Further research is needed to identify the different factors (e.g., social pressure, moral judgement) associated with the developmental patterns of GN‐based dehumanization.

#### 4.2.2 Developmental Trajectories of Blatant Versus Subtle Dehumanization

The current findings seem to suggest that blatant dehumanization emerged before subtle dehumanization and that the two did not share the same developmental trajectory. Indeed, it has been suggested that blatant and subtle forms of dehumanization are conceptually related but distinct mechanisms associated with different precedents and outcomes (Kteily et al. 2015). At first, these differing developmental trajectories of blatant and subtle dehumanization might seem surprising. Yet, an examination of other lines of relevant research, such as studies on racial bias, suggests that it is common to detect differing developmental patterns of explicit and implicit processes (Baron 2015; Qian et al. 2019; Raabe and Beelmann 2011). The differing trajectories in the present study may reflect a meaningful developmental sequence of blatant and subtle mechanisms. For instance, it is possible that conscious and explicit humanness perception about GN peers is formed earlier in childhood, but such blatant perception may remain unchallenged and may subsequently be normalized and internalized, contributing to the subsequent emergence of subtle dehumanization. Nevertheless, it is also plausible that the different onsets/trajectories of blatant and subtle dehumanization are partly due to the varying task demands and measurement properties of the two dehumanization tasks.

There has been very limited research examining simultaneously the developmental trajectories of both blatant and subtle dehumanization. Importantly, despite detecting imperfect alignment, the present study clearly shows that, by age 9–10 years, children both blatantly and subtly dehumanize peers who do not conform to gender norms. Further research is needed to corroborate the developmental trajectories of GN‐based dehumanization observed in the present study.

#### 4.2.3 Developing Link Between Dehumanization and Bullying

Interestingly, in the present study, increased bullying tendency for GN peers emerged before dehumanization of GN peers, implying that chronologically, dehumanization is not necessarily a developmental precursor of bullying. Yet, in the current sample, among older children, blatant dehumanization mediated the relationship between peer gender conformity and bullying tendency, suggesting that there may be a developmental change in the socio‐cognitive mechanisms underlying bullying of GN peers from early to late childhood and that dehumanization may be a driving force specific to older children. As children age, they may increasingly interpret GN behaviors of peers as indicative of other underlying traits, such as deviant character (Martin and Ruble 2010) and same‐sex attraction (Pascoe 2007; Plummer 2001). In this context, in older children, these other perceived underlying traits may add to the association between dehumanization and GN characteristics, facilitating punitive/bullying behaviors directed at dehumanized GN peers.

In the present study, there were mediating effects of blatant humanness ratings, but not mental state word proportions, on bullying tendency. These findings are in line with recent research proposing that blatant dehumanization is a relatively overt bias that motivates active aggression, while subtle dehumanization is commonly associated with passive indifference to the minds of others (Kteily and Landry 2022). In addition, it has also been suggested that self‐reported explicit measures may be more reliable and better predictors of behaviors compared with implicit measures, especially when behaviors are deliberately and explicitly measured (Corneille and Gawronski 2024; Dovidio et al. 2002). In the present study, only one explicit measure was used to assess bullying tendency, and blatant dehumanization was also assessed by an explicit measure, which may have contributed to the mediating effects of blatant dehumanization.

#### 4.2.4 Dehumanization of Norm Violators Versus Outgroup Members

Children in the present study did not consistently dehumanize all outgroup members based on gender; instead, children dehumanized GN peers regardless of their own gender and peer gender. Prior research has shown that gender norm violation is a highly salient characteristic that impacts children's peer judgements (Blakemore 2003; Carter and McCloskey 1984) and may override the influences of other social categories such as race on children's social evaluations (Qian et al. 2021).

Dehumanization is commonly believed to be directed at outgroups (Leyens et al. 2000), but it has been found that in adults, not all outgroups are perceived as less human‐like than ingroups (Bruneau, Jacoby, et al. 2018; Bruneau, Kteily, et al. 2018; Morehouse et al. 2023), and some outgroup characteristics and behaviors can moderate the degree of dehumanization (Jin et al. 2022; Kunst et al. 2019). Recent research on adults suggests that targets stigmatized as having lower status/socially disadvantaged (Bruneau, Jacoby, et al. 2018; Bruneau, Kteily, et al. 2018; Morehouse et al. 2023), immoral (Kteily and Landry 2022), or disrupting social order/norms (Fincher and Tetlock 2016; Rodríguez‐Gómez et al. 2022) may experience greater dehumanization than other targets. The current findings add to the growing research literature that calls for an intersectional approach to understanding social appraisals (Lei and Rhodes 2021) and dehumanization (Kteily and Landry 2022).

### 4.3 Limitations

The present study is not without limitations. First, only one explicit outcome measure was used to assess bullying tendency. Further research may employ explicit as well as implicit and spontaneous measures to assess different social outcomes such as prosocial and bullying tendencies.

Second, when assessing subtle dehumanization, the present study focused only on mind perception. Notably, subtle dehumanization is not limited to the denial of minds (Fincher et al. 2018) and may involve the denial of human‐typical traits and emotions (Haslam 2006; Leyens et al. 2000). In the present study, blatant dehumanization appeared to emerge before subtle dehumanization, but this observed sequence could have been partly attributable to the specific focus on mind perception assessment and the lack of assessment of other facets of subtle dehumanization. Future studies may develop child‐friendly and age‐appropriate tasks to assess children's attribution of human‐typical traits and emotions to GC and GN peers. Little is known about how children conceptualize humanness and how this conceptualization affects the link between blatant and subtle dehumanization in childhood. Recent research has paid increasing attention to these issues (Zhou et al. 2024), but further research using a wider range of dehumanization measures is needed to assess and establish the developmental patterns of both blatant and subtle dehumanization.

Third, the present study specifically focused on dehumanization but was not able to examine other mechanisms. Prior research has related children's perception of GN characteristics to children's gender essentialism (Gross et al. 2024) and parents’ social‐political views (Foster‐Hanson and Rhodes 2022). Further research may examine how different mechanisms may act together to shape children's GN‐based hostility.

Fourth, children's liking of peers was not measured or controlled for. One possibility is that children's dehumanization of GN peers and their association with bullying may be explained by their dislike of GN peers. Nevertheless, it has been found that dehumanization can independently predict hostility after controlling for liking (Goff et al. 2008; Kteily et al. 2015). There is also evidence supporting a double dissociation in neural underpinnings between dehumanization and liking (Bruneau, Jacoby, et al. 2018; Bruneau, Kteily, et al. 2018). Still, further research may simultaneously include liking and dehumanization measures to examine their relative contributions to children's evaluations and behavioral treatment of GN peers.

Finally, inevitably, experimental manipulations and vignettes of hypothetical peers have limited ecological validity. However, conducting field research may involve identifying GN peers in classes or peer groups and may increase unwanted attention directed to the GN peers in real‐life settings. Therefore, although the current peer vignettes have their limitations, they enable effective, standardized, and neat manipulations of peer gender (non)conformity in an experimental setting, contributing to a greater understanding of the causal relationship between peer gender (non)conformity and dehumanization.

### 4.4 Implications

Hostility toward gender/sexual minorities may have its developmental roots in GN‐based dehumanization in childhood. Gender nonconformity is commonly (mis)perceived as a sign of same‐sex attraction (Henry and Steiger 2022) and transgender identity (Howansky et al. 2020). Across cultures, gender/sexual minorities might be addressed using dehumanizing language in daily life conversations (Fasoli et al. 2016; Poteat et al. 2012) and mainstream media (Mendelsohn et al. 2020; Taha‐Thomure et al. 2022). Specifically, in Hong Kong, gender/sexual minorities are sometimes described as non‐humans, such as monsters, ghosts, and the dead (Wong and Cheung 2018). Timely interventions designed to tackle children's GN‐based dehumanization may help reduce developing individuals’ hostility against gender/sexual minorities beyond childhood.

Although it has been well documented that GN individuals experience long‐term negative psychosocial outcomes associated with childhood rejection and victimization (Narita et al. 2024; Roberts et al. 2013), interventions targeting a specific developmental stage or socio‐cognitive mechanism are lacking. Timely interventions aimed at reducing children's dehumanization of GN peers may be useful in reducing GN‐based bullying and hostility. Further research may usefully examine whether interventions based on existing techniques, such as reasserting human‐typical traits (Howe et al. 2022), prompted mentalization (McLoughlin and Over 2019), and imagined contact (Prati and Loughnan 2018; Vezzali et al. 2012), are effective in rehumanizing appraisals of GN peers and reducing GN‐based bullying among children.

## 5 Conclusion

The present study was the first to examine GN‐based dehumanization in children and its developmental trajectory, revealing that by age 9–10 years, children both blatantly and subtly dehumanize GN peers. These findings make a unique contribution to the literature by identifying dehumanization as a potential socio‐cognitive mechanism underlying children's hostility toward GN peers. The mediating effects of dehumanization on bullying varied according to the form of dehumanization. Further research may employ a wider range of outcome measures assessing bullying and prosociality to provide additional insights into the links between different forms of dehumanization and treatment of GN peers. Further research is needed to develop and evaluate interventions designed to reduce GN‐based dehumanization in children.

## Author Contributions

M.M.C.H. and K.T.F.K. designed the study. M.M.C.H. conducted the study and analyzed the data under the supervision of K.T.F.K. M.M.C.H. wrote the paper with input and critical revisions from K.T.F.K.

## Ethics Statement

The study was approved by the Human Research Ethics Committee at the University of Hong Kong.

## Conflicts of Interest

The authors declare no conflicts of interest.

## Permission to Reproduce Material From Other Sources

License to reuse the insect scale image was obtained from Elsevier (license number: 5966820632671).

## Supporting information




**Supporting File 1**: desc70070‐sup‐0001‐SuppMat.docx

## Data Availability

Anonymized data used for the reported analyses are available from the authors upon reasonable request. The syntax and outputs of statistical analyses are also available from the authors upon reasonable request.
