# Supplementary material for: Experimental Evidence of Peer Gender Nonconformity Triggering Dehumanization in Children: Developmental Trajectory, Form, and Link to Bullying
Source: Dev Sci. 2025 Sep 5;28(6):e70070. doi: 10.1111/desc.70070 (PMC12412079; doi:10.1111/desc.70070)
Supplement: Supplementary file 1 — Supporting File 1: desc70070‐sup‐0001‐SuppMat.docx [file DESC-28-e70070-s001.docx]

**SUPPORTING INFORMATION**

Hui, M. M. C., & Kung, K. T. F. Experimental evidence of peer gender nonconformity triggering dehumanization in children: Developmental trajectory, form, and link to bullying. *Developmental Science*

1. **Peer vignette samples**

| 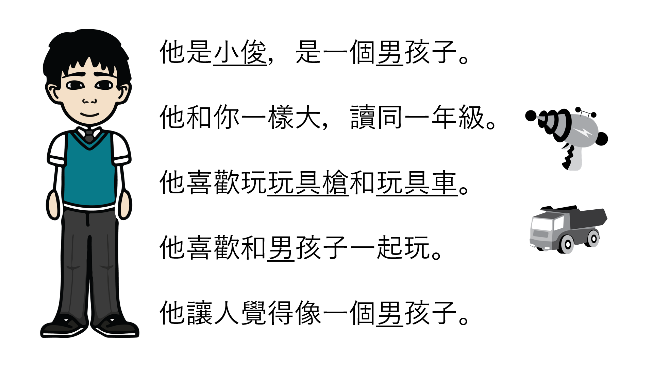 | 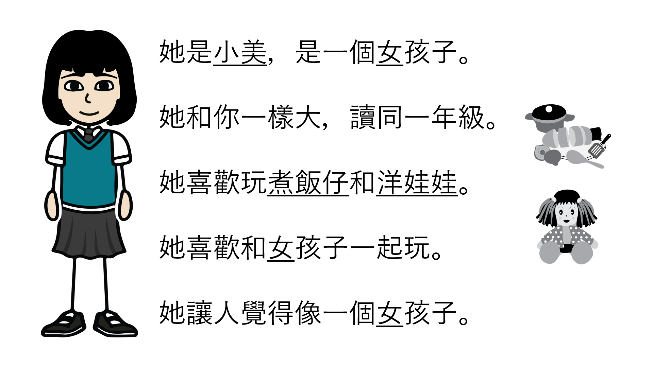 |
| --- | --- |
| **Gender conforming (GC) boy vignette sample**  English translation:  He is Siu Chun (a boy-typical name).  He is a boy.  He is your age and in your grade.  He likes playing with toy guns and toy trucks.  He likes playing with boys.  People think he is boyish. | **Gender conforming (GC) girl vignette sample**  English translation:  She is Siu Mei (a girl-typical name).  She is a girl.  She is your age and in your grade.  She likes playing with kitchen sets and dolls.  She likes playing with girls.  People think she is girlish. |
| 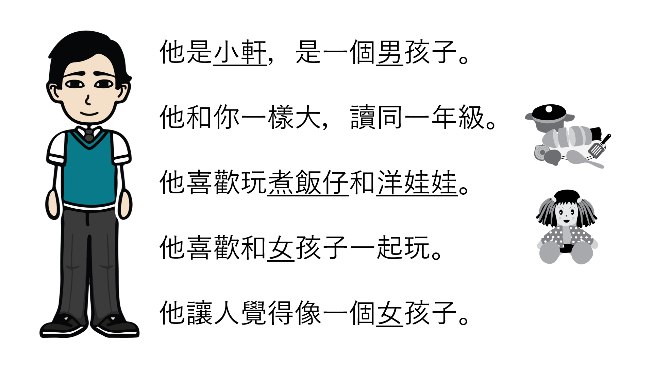 | 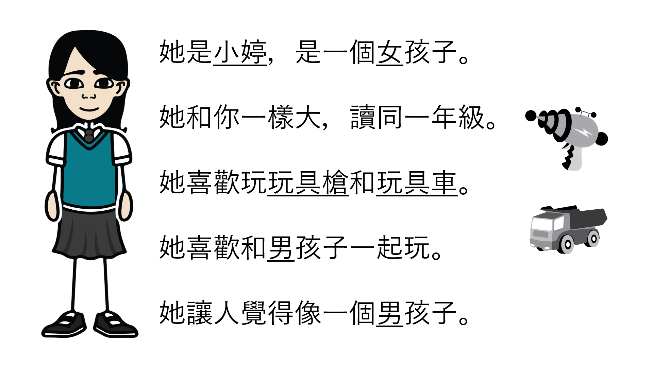 |
| **Gender nonconforming (GN) boy vignette sample**  English translation:  He is Siu Hin (a boy-typical name).  He is a boy.  He is your age and in your grade.  He likes playing with kitchen sets and dolls.  He likes playing with girls.  People think he is girlish. | **Gender nonconforming (GN) girl vignette sample**  English translation:  She is Siu Ting (a girl-typical name).  She is a girl.  She is your age and in your grade.  She likes playing with toy guns and toy trucks.  She likes playing with boys.  People think she is boyish. |

1. **Mental state word coding scheme**

Mental state words were counted only when their usage referred to the animation characters’ mental states (e.g., “He thinks…”), but not the participant’s mental states (e.g., “I think…”). Immediate repetition of mental state words (e.g., stuttering, self-correction) was only counted once.

| **Categories** | **Mental state word examples** |
| --- | --- |
| Desire and Intention | Desire, Love/Like, Dislike/Hate, Want, Favorite, Need, Wish, Willing to, Determined to, Try to, Intend to, Dare to, Purposefully, Work hard, Give up |
| Emotions | Emotion, Feel, Happy, Excited, Angry, Sad/ Upset, Frightened/ Scared, Surprised, Startled, Worried, Concern/ Worry, Bored, Grateful/ Appreciative, Anxious, Disappointed, Amused, Relaxed, Uncomfortable, Aggrieved, Embarrassed, Hatred, Lonely, Grumpy, Jealous, Annoyed, Proud, Regret, Helpless, Disgusted, Calm, Stressed |
| Cognitions | Think, Falsely believe, Know, Who knows, It turns out, Remember, Guess/ Bet/ Assume, Suspect, Question, Smart/ intelligent, Stupid, Discover/ Realize, Understand, Concentrate/ Focus, Pay attention, Notice/ Aware, Carefully/ Carelessly, Forgive, Let go, Expect, Daydreaming/ Distracted, Learn, Thoughts, Admit, Alert, Accept, Agree, Opinion, Reflect, Consider, Tolerate, Promise, Despise, Admire, Value, Plan, Decide |
| Actions that involve interactions of mental states | Tricking, Teasing/ Bullying, Arguing, Joking, Taunting, Harassing/ Annoying, Telling off, Breaking up, Reconciling, Apologizing, Surprising, Spying, Comforting, Caring, Helping, Saving, Encouraging, Persuading, Luring, Praising, Lying/ Cheating, Pretending |
| Personality traits | Personality, Careless, Impulsive, Timid, Brave, Tough, Naughty, Well-behaved, Playful, Friendly, Polite, Rude/ Despicable, Innocent, Selfish, Curious, Stingy, Introverted, Violent, Cunning, Proactive, Bossy, Enthusiastic, Generous, Mature, Considerate, Optimistic, Open-minded, Bothersome, Confident |

1. **Descriptive statistics**

| **Participant Gender** | **Age Group** | **Peer Gender** | **Peer Gender Conformity** | **Blatant Humanness Rating**  **(*n* = 444)** | | **Frequency Proportion**  **(*n* = 440)** | | **Diversity Proportion**  **(*n* = 435)** | | **Bullying Tendency**  **(*n* = 449)** | |
| --- | --- | --- | --- | --- | --- | --- | --- | --- | --- | --- | --- |
|  |  |  |  | *M* | *SD* | *M* | *SD* | *M* | *SD* | *M* | *SD* |
| Boy | 5-6y | Boy | GC | 81.2 | 26.8 | .019 | .028 | .014 | .018 | 1.5 | 0.7 |
|  |  |  | GN | 76.7 | 28.0 | .019 | .026 | .010 | .013 | 1.8 | 0.9 |
|  |  | Girl | GC | 78.2 | 24.0 | .020 | .029 | .011 | .015 | 1.7 | 0.8 |
|  |  |  | GN | 74.5 | 24.4 | .017 | .027 | .010 | .013 | 1.9 | 1.0 |
|  | 7-8y | Boy | GC | 81.2 | 25.3 | .023 | .020 | .016 | .016 | 1.3 | 0.6 |
|  |  |  | GN | 74.3 | 23.7 | .027 | .027 | .018 | .017 | 1.4 | 0.7 |
|  |  | Girl | GC | 84.5 | 22.8 | .021 | .023 | .015 | .017 | 1.3 | 0.6 |
|  |  |  | GN | 72.4 | 24.1 | .023 | .023 | .018 | .018 | 1.4 | 0.7 |
|  | 9-10y | Boy | GC | 88.2 | 17.3 | .032 | .024 | .022 | .017 | 1.2 | 0.4 |
|  |  |  | GN | 75.2 | 25.4 | .027 | .020 | .017 | .013 | 1.5 | 0.7 |
|  |  | Girl | GC | 83.2 | 19.8 | .036 | .025 | .023 | .016 | 1.3 | 0.4 |
|  |  |  | GN | 78.4 | 20.4 | .031 | .022 | .020 | .013 | 1.4 | 0.6 |
|  | 11-12y | Boy | GC | 87.1 | 18.4 | .036 | .026 | .024 | .017 | 1.1 | 0.3 |
|  |  |  | GN | 71.6 | 26.9 | .030 | .024 | .019 | .015 | 1.5 | 0.7 |
|  |  | Girl | GC | 90.0 | 14.0 | .039 | .027 | .028 | .018 | 1.2 | 0.5 |
|  |  |  | GN | 77.6 | 21.9 | .031 | .021 | .019 | .012 | 1.4 | 0.7 |
| Girl | 5-6y | Boy | GC | 78.1 | 21.9 | .020 | .028 | .015 | .020 | 1.5 | 0.6 |
|  |  |  | GN | 81.7 | 22.8 | .019 | .023 | .014 | .015 | 1.4 | 0.6 |
|  |  | Girl | GC | 80.4 | 26.7 | .021 | .024 | .012 | .017 | 1.3 | 0.5 |
|  |  |  | GN | 78.4 | 29.9 | .019 | .022 | .017 | .020 | 1.6 | 0.7 |
|  | 7-8y | Boy | GC | 82.6 | 23.1 | .025 | .026 | .017 | .016 | 1.4 | 0.6 |
|  |  |  | GN | 73.2 | 24.9 | .030 | .029 | .017 | .017 | 1.5 | 0.7 |
|  |  | Girl | GC | 87.3 | 20.5 | .027 | .029 | .014 | .014 | 1.4 | 0.6 |
|  |  |  | GN | 76.9 | 26.4 | .026 | .023 | .017 | .014 | 1.5 | 0.8 |
|  | 9-10y | Boy | GC | 88.4 | 16.1 | .033 | .027 | .022 | .018 | 1.2 | 0.3 |
|  |  |  | GN | 72.6 | 22.0 | .027 | .021 | .017 | .013 | 1.4 | 0.6 |
|  |  | Girl | GC | 89.6 | 17.7 | .036 | .026 | .024 | .016 | 1.1 | 0.3 |
|  |  |  | GN | 74.3 | 20.0 | .033 | .023 | .022 | .014 | 1.4 | 0.5 |
|  | 11-12y | Boy | GC | 85.6 | 20.2 | .034 | .027 | .020 | .014 | 1.2 | 0.4 |
|  |  |  | GN | 72.6 | 22.4 | .036 | .024 | .022 | .015 | 1.5 | 0.7 |
|  |  | Girl | GC | 88.1 | 20.7 | .036 | .023 | .025 | .015 | 1.2 | 0.4 |
|  |  |  | GN | 74.4 | 23.4 | .032 | .024 | .021 | .016 | 1.4 | 0.5 |

**Remarks. GC = Gender conforming; GN = Gender nonconforming**

1. **Parametric analyses**
2. **Parametric ANOVA table on blatant humanness rating (*N* = 444)**

| **Variables** | ***F*** | ***p*** | ***η_p_^2^*** |
| --- | --- | --- | --- |
| Peer Gender | 1.89 | .169 | .00 |
| Peer Gender x Age Group | 1.74 | .158 | .01 |
| Peer Gender x Participant Gender | 0.73 | .395 | .00 |
| Peer Gender x Age Group x Participant Gender | 0.62 | .605 | .00 |
| Peer Gender Conformity | 79.86 | < .001 | .15 |
| Peer Gender Conformity x Age Group | 6.45 | < .001 | .04 |
| Peer Gender Conformity x Participant Gender | 0.03 | .861 | .00 |
| Peer Gender Conformity x Age Group x Participant Gender | 1.30 | .273 | .01 |
| Peer Gender x Peer Gender Conformity | 0.00 | .996 | .00 |
| Peer Gender x Peer Gender Conformity x Age Group | 1.02 | .383 | .01 |
| Peer Gender x Peer Gender Conformity x Participant Gender | 0.98 | .324 | .00 |
| Peer Gender x Peer Gender Conformity x Age Group x Participant Gender | 0.61 | .607 | .00 |
| Age Group | 0.77 | .514 | .01 |
| Participant Gender | 0.16 | .685 | .00 |
| Age Group x Participant Gender | 0.32 | .814 | .00 |

Parametric ANOVA revealed a significant main effect of peer gender conformity, *F*(1, 436) = 79.86, *p* < .001, η_p_^2^ = .15, and a significant peer gender conformity x age group interaction, *F*(3, 436) = 6.45, *p* < .001, η_p_^2^ = .04.

Parametric post hoc analyses were conducted to follow up the peer gender conformity x age group interaction. Children in the 5-6y age group did not rate GN peers (*M* = 77.8, *SD* = 20.7) and GC peers (*M* = 79.5, *SD* = 17.2) differently, *M*_diff_ = −1.6, 95% CI [−5.85, 2.63], *p* = .456, *d* = −0.07. Children in the 7-8y age group rated GN peers (*M* = 74.2, *SD* = 20.7) as less human-like/ more insect-like than GC peers (*M* = 83.9, *SD* = 17.2), *M*_diff_ = −9.7, 95% CI [−13.83, −5.59], *p* < .001, *d* = −0.48. Children in the 9-10y age group also rated GN peers (*M* = 75.1, *SD* = 20.6) as less human-like/ more insect-like than GC peers (*M* = 87.4, *SD* = 17.2), *M*_diff_ = −12.2, 95% CI [−16.24, −8.18], *p* < .001, *d* = −0.71. Lastly, children in the 11-12y age group rated GN peers (*M* = 74.1, *SD* = 20.7) as less human-like/ more insect-like than GC peers (*M* = 87.7, *SD* = 17.2), *M*_diff_ = −13.6, 95% CI [−17.60, −9.68], *p* < .001, *d* = −0.74. Compared to the 5-6y age group, the differences in ratings between GC and GN peers were significantly greater in the 7-8y age group, *M*_diff_ = 8.2, 95% CI [2.32, 14.14], *p* = .006, *d* = 0.37, the 9-10y age group, *M*_diff_ = 10.8, 95% CI [4.93, 16.62], *p* < .001, *d* = 0.51, and the 11-12y age group, *M*_diff_ = 12.1, 95% CI [6.35, 17.95], *p* < .001, *d* = 0.58.

1. **Parametric ANOVA table on frequency proportion of mental state words (*N* = 440)**

| **Variables** | ***F*** | ***p*** | ***η_p_^2^*** |
| --- | --- | --- | --- |
| Peer Gender | 0.46 | .499 | .00 |
| Peer Gender x Age Group | 1.60 | .188 | .01 |
| Peer Gender x Participant Gender | 0.04 | .841 | .00 |
| Peer Gender x Age Group x Participant Gender | 0.22 | .883 | .00 |
| Peer Gender Conformity | 4.02 | .046 | .01 |
| Peer Gender Conformity x Age Group | 3.10 | .027 | .02 |
| Peer Gender Conformity x Participant Gender | 0.60 | .438 | .00 |
| Peer Gender Conformity x Age Group x Participant Gender | 0.69 | .561 | .00 |
| Peer Gender x Peer Gender Conformity | 1.21 | .272 | .00 |
| Peer Gender x Peer Gender Conformity x Age Group | 0.46 | .712 | .00 |
| Peer Gender x Peer Gender Conformity x Participant Gender | 0.02 | .885 | .00 |
| Peer Gender x Peer Gender Conformity x Age Group x Participant Gender | 0.13 | .945 | .00 |
| Age Group | 17.64 | < .001 | .11 |
| Participant Gender | 0.87 | .351 | .00 |
| Age Group x Participant Gender | 0.21 | .891 | .00 |

Parametric ANOVA showed a significant main effect of age group, *F*(3, 432) = 17.64, *p* < .001, η_p_^2^ = .11, a significant main effect of peer gender conformity, *F*(1, 432) = 4.02, *p* = .046, η_p_^2^ = .01, and a significant peer gender conformity x age group interaction, *F*(3, 432) = 3.10, *p* = .027, η_p_^2^ = .02. All other main effects and interactions were non-significant.

Regarding the main effect of age group, older children generally produced a higher frequency proportion of mental state words than did younger children. Children in the 7-8y age group (*M* = .025, *SD* = .017) produced more mental state words than did children in the 5-6y age group (*M* = .019, *SD* = .017), *M*_diff_ = .006, 95% CI [.001, .010], *p* = .009, *d* = 0.35; children in the 9-10y age group (*M* = .032, *SD* = .017) produced more mental state words than did children in the 7-8y age group, *M*_diff_ = .007, 95% CI [.002, .011], *p* = .003, *d* = 0.40. However, children in the 11-12y age group (*M* = .034, *SD* = .017) did not produce more mental state words than did children in the 9-10y age group, *M*_diff_ = .002, 95% CI [−.002, .007], *p* = .280, *d* = 0.15.

Planned post hoc analyses were conducted to follow up the peer gender conformity x age group interaction. Children in the 5-6y age group did not differ in mental state word frequency proportion when describing GN peers (*M* = .019, *SD* = .019) and GC peers (*M* = .020, *SD* = .020), *M*_diff_ = −.001, 95% CI [−.005, .003], *p* = .552, *d* = −0.06. Children in the 7-8y age group also did not differ in mental state word frequency proportion when describing GN peers (*M* = .027, *SD* = .019) and GC peers (*M* = .024, *SD* = .020), *M*_diff_ = .003, 95% CI [−.001, .006], *p* = .191, *d* = 0.13. Nevertheless, children in the 9-10y age group used fewer mental state words to describe GN peers (*M* = .029, *SD* = .019) than to describe GC peers (*M* = .034, *SD* = .020), *M*_diff_ = −.005, 95% CI [−.009, −.001], *p* = .011, *d* = −0.26. Children in the 11-12y age group also used fewer mental state words to describe GN peers (*M* = .032, *SD* = .019) than to describe GC peers (*M* = .036, *SD* = .020), *M*_diff_ = −.004, 95% CI [−.008, −.0005], *p* = .028, *d* = −0.23. The differences in mental state word frequency proportion between GC and GN peers in the 9-10y and 11-12y age groups were greater than those in the 7-8y age group (9-10y > 7-8y: *M*_diff_ = .007, 95% CI [.002, .013], *p* = .007, *d* = 0.35; 11-12y > 7-8y: *M*_diff_ = .007, 95% CI [.001, .012], *p* = .014, *d* = 0.34).

1. **Parametric ANOVA table on diversity proportion of mental state words (*N* = 435)**

| **Variables** | ***F*** | ***p*** | ***η_p_^2^*** |
| --- | --- | --- | --- |
| Peer Gender | 1.16 | .281 | .00 |
| Peer Gender x Age Group | 1.90 | .128 | .01 |
| Peer Gender x Participant Gender | 0.38 | .537 | .00 |
| Peer Gender x Age Group x Participant Gender | 0.17 | .914 | .00 |
| Peer Gender Conformity | 6.15 | .014 | .01 |
| Peer Gender Conformity x Age Group | 4.90 | .002 | .03 |
| Peer Gender Conformity x Participant Gender | 2.78 | .096 | .01 |
| Peer Gender Conformity x Age Group x Participant Gender | 1.25 | .291 | .01 |
| Peer Gender x Peer Gender Conformity | 1.04 | .308 | .00 |
| Peer Gender x Peer Gender Conformity x Age Group | 2.41 | .067 | .02 |
| Peer Gender x Peer Gender Conformity x Participant Gender | 0.36 | .550 | .00 |
| Peer Gender x Peer Gender Conformity x Age Group x Participant Gender | 0.09 | .965 | .00 |
| Age Group | 16.01 | < .001 | .10 |
| Participant Gender | 0.46 | .498 | .00 |
| Age Group x Participant Gender | 0.60 | .614 | .00 |

Parametric ANOVA showed a significant main effect of age group, *F*(3, 427) = 16.01, *p* < .001, η_p_^2^ = .10, a significant main effect of peer gender conformity, *F*(1, 427) = 6.15, *p* = .014, η_p_^2^ = .01, and a significant peer gender conformity x age group interaction, *F*(3, 427) = 4.90, *p* = .002, η_p_^2^ = .03. All other main effects and interactions were non-significant.

Regarding the main effect of age group, older children generally produced a higher diversity proportion of mental state words than did younger children when describing peers. Children in the 7-8y age group (*M* = .017, *SD* = .011) produced more diverse mental state words than did children in the 5-6y age group (*M* = .013, *SD* = .011), *M*_diff_ = .004, 95% CI [.001, .007], *p* = .016, *d* = 0.31; children in the 9-10y age group (*M* = .021, *SD* = .011) produced more diverse mental state words than did children in the 7-8y age group, *M*_diff_ = .004, 95% CI [.001, .007], *p* = .005, *d* = 0.39. However, children in the 11-12y age group (*M* = .022, *SD* = .011) did not produce more diverse mental state words than did children in the 9-10y age group, *M*_diff_ = .001, 95% CI [−.001, .004], *p* = .335, *d* = 0.14.

Planned post hoc analyses were conducted to follow up the peer gender conformity x age group interaction. Children in the 5-6y age group did not differ in mental state word diversity proportion when describing GN peers (*M* = .013, *SD* = .012) and GC peers (*M* = .013, *SD* = .013), *M*_diff_ = −.001, 95% CI [−.003, .002], *p* = .659, *d* = −0.04. Children in the 7-8y age group also did not differ in mental state word diversity proportion when describing GN peers (*M* = .018, *SD* = .012) and GC peers (*M* = .016, *SD* = .013), *M*_diff_ = .002, 95% CI [−.001, .004], *p* = .129, *d* = 0.14. Nevertheless, children in the 9-10y age group used less diverse mental state words when describing GN peers (*M* = .019, *SD* = .012) than when describing GC peers (*M* = .023, *SD* = .013), *M*_diff_ = −.004, 95% CI [−.006, −.001], *p* = .002, *d* = −0.32. Children in the 11-12y age group also used less diverse mental state words when describing GN peers (*M* = .020, *SD* = .012) than when describing GC peers (*M* = .024, *SD* = .013), *M*_diff_ = −.004, 95% CI [−.006, −.001], *p* = .003; *d* = −0.30. The differences in mental state word diversity proportion between GC and GN peers in the 9-10y and 11-12y age groups were significantly greater than those in the 7-8y age group (9-10y > 7-8y: *M*_diff_ = .006, 95% CI [.002, .009], *p* = .001, *d* = 0.43; 11-12y > 7-8y: *M*_diff_ = .006, 95% CI [.002, .009], *p* = .002, *d* = 0.45).

1. **Parametric ANOVA table on bullying tendency scores (*N* = 449)**

| **Variables** | ***F*** | ***p*** | ***η_p_^2^*** |
| --- | --- | --- | --- |
| Peer Gender | 0.05 | .819 | .00 |
| Peer Gender x Age Group | 1.61 | .187 | .01 |
| Peer Gender x Participant Gender | 2.20 | .138 | .00 |
| Peer Gender x Age Group x Participant Gender | 0.82 | .482 | .01 |
| Peer Gender Conformity | 60.17 | < .001 | .12 |
| Peer Gender Conformity x Age Group | 1.23 | .299 | .01 |
| Peer Gender Conformity x Participant Gender | 0.68 | .410 | .00 |
| Peer Gender Conformity x Age Group x Participant Gender | 0.60 | .613 | .00 |
| Peer Gender x Peer Gender Conformity | 2.78 | .096 | .01 |
| Peer Gender x Peer Gender Conformity x Age Group | 1.91 | .127 | .01 |
| Peer Gender x Peer Gender Conformity x Participant Gender | 6.65 | .010 | .01 |
| Peer Gender x Peer Gender Conformity x Age Group x Participant Gender | 1.13 | .335 | .01 |
| Age Group | 8.03 | < .001 | .05 |
| Participant Gender | 3.31 | .070 | .01 |
| Age Group x Participant Gender | 2.91 | .034 | .02 |

Parametric ANOVA showed a significant main effect of peer gender conformity, *F*(1, 441) = 60.17, *p* < .001, η_p_^2^ = .12, a significant main effect of age group, *F*(3, 441) = 8.03, *p* < .001, η_p_^2^ = .05, a significant age group x participant gender interaction, *F*(3, 441) = 2.91, *p* = .034, η_p_^2^ = .02, and a significant peer gender x peer gender conformity x participant gender interaction, *F*(1, 441) = 6.65, *p* = .010, η_p_^2^ = .01.

Regarding the main effect of age group, participants aged 5-6y (*M* = 1.6, *SD* = 0.5) reported significantly higher bullying tendency towards hypothetical peers than participants aged 7-8y (*M* = 1.4, *SD* = 0.5), *M*_diff_ = 0.2, 95% CI [0.06, 0.32], *p* = .004, *d* = 0.34, participants aged 9-10y (*M* = 1.3, *SD* = 0.5), *M*_diff_ = 0.3, 95% CI [0.15, 0.41], *p* < .001, *d* = 0.56, and participants aged 11-12y (*M* = 1.3, *SD* = 0.5), *M*_diff_ = 0.3, 95% CI [0.15, 0.41], *p* < .001, *d* = 0.54. Other differences between age groups were non-significant.

Regarding the age group x participant gender interaction, for the 5-6y age group, boy participants (*M* = 1.8, *SD* = 0.5) reported a higher bullying tendency towards hypothetical peers than girl participants (*M* = 1.4, *SD* = 0.5), *M*_diff_ = 0.3, 95% CI [0.13, 0.50], *p* < .001, *d* = 0.54. However, boy and girl participants reported similar levels of bullying tendency in the 7-8y age group, *M*_diff_ = −0.1, 95% CI [−0.25, 0.13], *p* = .527, *d* = −0.11, the 9-10y age group, *M*_diff_ = 0.1, 95% CI [−0.11, 0.25], *p* = .437, *d* = 0.18, and the 11-12y age group, *M*_diff_ = 0, 95% CI [−0.16, 0.19], *p* = .876, *d* = 0.03.

To investigate the peer gender x peer gender conformity x participant gender interaction, peer gender x participant gender ANOVAs were conducted separately for each gender conformity condition. For GC peers, the main effect of peer gender, *F*(1, 447) = 2.19, *p* = .140, η_p_^2^ = .00, and the main effect of participant gender, *F*(1, 447) = 1.89, *p* = .170, η_p_^2^ = .00, were non-significant. The peer gender x participant gender interaction was significant, *F*(1, 447) = 9.27, *p* = .002, η_p_^2^ = .02. While boy participants reported significantly lower bullying tendency towards GC boys (*M* = 1.3, *SD* = 0.5) than towards GC girls (*M* = 1.4, *SD* = 0.6), *M*_diff_ = −0.1, 95% CI [−0.17, −0.04], *p* = .002, *d* = −0.18; girl participants reported similar bullying tendency towards GC boys (*M* = 1.3, *SD* = 0.5) and GC girls (*M* = 1.3, *SD* = 0.5), *M*_diff_ = 0, 95% CI [−0.03, 0.10], *p* = .265, *d* = 0.08. For GN peers, the main effect of peer gender, *F*(1, 447) = 1.06, *p* = .304, η_p_^2^ = .00, and the main effect of participant gender, *F*(1, 447) = 2.82, *p* = .094, η_p_^2^ = .01, were both non-significant. The peer gender x participant gender interaction was also non-significant, *F*(1, 447) = 0.50, *p* = .481, η_p_^2^ = .00. In other words, participants’ bullying tendency towards GN peers did not depend on peer gender or participant gender. Participants consistently reported a higher tendency to bully GN peers than GC peers.

1. **Nonparametric analyses**

For nonparametric analyses, we first applied the Aligned Rank Transform (ART) procedures to convert data into aligned ranks using the ARTool software package (Wobbrock et al., 2011). Contrary to traditional nonparametric tests, ART allows nonparametric analyses of interaction effects in models with multiple factors. Aligned ranks were then applied to the mixed ANOVA analyses in SPSS. Planned post hoc Wilcoxon signed rank tests (for within-subject factors) and Mann-Whitney tests/Kruskal-Wallis tests (for between-subject factors) were conducted to follow up on significant interaction effects in nonparametric ANOVA. Effect sizes of nonparametric post hoc analyses were estimated using r (small: .10; medium: .30; large: .50) or η^2^ (small: .01; medium: .06; large: .14) (Cohen, 1988).

1. **Nonparametric ANOVA table on blatant humanness rating (*N* = 444)**

| **Variables** | ***F*** | ***p*** | ***η_p_^2^*** |
| --- | --- | --- | --- |
| Peer Gender | 1.89 | .170 | .00 |
| Peer Gender x Age Group | 1.08 | .358 | .01 |
| Peer Gender x Participant Gender | 5.78 | .017 | .01 |
| Peer Gender x Age Group x Participant Gender | 1.32 | .267 | .01 |
| Peer Gender Conformity | 94.20 | < .001 | .18 |
| Peer Gender Conformity x Age Group | 5.49 | .001 | .04 |
| Peer Gender Conformity x Participant Gender | 0.02 | .887 | .000 |
| Peer Gender Conformity x Age Group x Participant Gender | 2.69 | .046 | .02 |
| Peer Gender x Peer Gender Conformity | 0.05 | .824 | .00 |
| Peer Gender x Peer Gender Conformity x Age Group | 0.75 | .522 | .01 |
| Peer Gender x Peer Gender Conformity x Participant Gender | 0.02 | .877 | .00 |
| Peer Gender x Peer Gender Conformity x Age Group x Participant Gender | 1.10 | .350 | .01 |
| Age Group | 0.30 | .822 | .00 |
| Participant Gender | 0.12 | .731 | .00 |
| Age Group x Participant Gender | 0.43 | .730 | .00 |

Nonparametric ANOVA on blatant humanness ratings was conducted. Consistent with parametric analyses, the main effect of peer gender conformity, *F*(1, 436) = 94.20, *p* < .001, η_p_^2^ = .18, and the peer gender conformity x age group interaction, *F*(3, 436) = 5.49, *p* = .001, η_p_^2^ = .04, were significant.

Nonparametric post hoc analyses were conducted to follow up on the peer gender conformity x age group interaction. Results were consistent with those from parametric post hoc analyses. Children in the 5-6y age group did not rate GN peers and GC peers differently (Wilcoxon signed rank test: *Z* = −0.63, *p* = .528, *r* = .06). Children in the 7-8y age group rated GN peers as less human-like/ more insect-like than GC peers (Wilcoxon signed rank test: *Z* = −4.07, *p* < .001, *r* = .39)*.* Children in the 9-10y age group also rated GN peers as less human-like/ more insect-like than GC peers (Wilcoxon signed rank test: *Z* = −5.66, *p* < .001, *r* = .53). Lastly, children in the 11-12y age group rated GN peers as less human-like/ more insect-like than GC peers (Wilcoxon signed rank test: *Z* = −6.29, *p* < .001, *r* = .58).

In nonparametric analyses, there were a significant peer gender conformity x age group x participant gender interaction and a significant peer gender x participant gender interaction. Both older boy and girl participants tended to rate GC peers as more human-like/less insect-like than did younger participants (for boy participants, Kruskal-Wallis test: *H*(3) = 6.03, *p* = .110, η^2^ = .02; for girl participants, Kruskal-Wallis test: *H*(3) = 10.44, *p* = .015, η^2^ = .04), but it appeared that only in girls where older participants gave less human-like/more insect-like ratings to GN peers than did younger participants (for boy participants, Kruskal-Wallis test: *H*(3) = 0.81, *p* = .847, η^2^ = .01; for girl participants, Kruskal-Wallis test: *H*(3) = 6.37, *p* = .095, η^2^ = .02). While it is clear that both older boys and girls dehumanize GN peers, some inconsistent patterns or weak trends seem to suggest that older girls’ GC-GN differences in ratings were driven by both their higher ratings for GC peers and lower ratings for GN peers and that older boys’ GC-GN differences in ratings were driven mainly by their higher ratings for GC peers and less by their lower ratings for GN peers. In addition, post hoc tests revealed an overall pattern for girl participants to rate boy peers as less human-like/more insect-like than girl peers (Wilcoxon signed rank test: *Z* = 2.44, *p* = .015, *r* = .16), but no differences in boy participants’ ratings for boy versus girl peers (Wilcoxon signed rank test: *Z* = 0.14, *p =* .890, *r* = .01). However, this pattern for girls to rate boy and girl peers differently was not significant or consistent across age groups. The strongest effect for this was observed in girls’ ratings for boy versus girl peers in the 7-8y age group, but even this strongest effect did not reach statistical significance (Wilcoxon signed rank test: *Z* = 1.88, *p* = .059, *r* = .25). Hence, although it is clear that older children dehumanize GN peers, there is no consistent or robust pattern suggesting that children dehumanize peers simply based on children’s own gender or peers’ gender.

1. **Nonparametric ANOVA table on frequency proportion of mental state words (*N* = 440)**

| **Variables** | ***F*** | ***p*** | ***η_p_^2^*** |
| --- | --- | --- | --- |
| Peer Gender | 1.46 | .228 | .00 |
| Peer Gender x Age Group | 1.94 | .122 | .01 |
| Peer Gender x Participant Gender | 0.30 | .586 | .00 |
| Peer Gender x Age Group x Participant Gender | 0.32 | .811 | .00 |
| Peer Gender Conformity | 2.65 | .105 | .01 |
| Peer Gender Conformity x Age Group | 3.21 | .023 | .02 |
| Peer Gender Conformity x Participant Gender | 1.89 | .170 | .00 |
| Peer Gender Conformity x Age Group x Participant Gender | 0.57 | .636 | .00 |
| Peer Gender x Peer Gender Conformity | 0.72 | .397 | .00 |
| Peer Gender x Peer Gender Conformity x Age Group | 0.39 | .761 | .00 |
| Peer Gender x Peer Gender Conformity x Participant Gender | 0.41 | .520 | .00 |
| Peer Gender x Peer Gender Conformity x Age Group x Participant Gender | 0.36 | .779 | .00 |
| Age Group | 24.91 | < .001 | .15 |
| Participant Gender | 1.16 | .282 | .00 |
| Age Group x Participant Gender | 0.07 | .977 | .00 |

Nonparametric results were highly similar to parametric results. Nonparametric analyses showed a significant main effect of age group, *F*(3, 432) = 24.91, *p* < .001, η_p_^2^ = .15, and a significant peer gender conformity x age group interaction, *F*(3, 432) = 3.21 *p* = .023, η_p_^2^ = .02.

Regarding the main effect of age group, older children generally produced a higher frequency proportion of mental state words than did younger children. Children in the 7-8y age group produced more mental state words than did children in the 5-6y age group (Mann-Whitney test: *Z =* 3.01, *p* = .003, *r* = .20); children in the 9-10y age group produced more mental state words than did children in the 7-8y age group (Mann-Whitney test: *Z* = 3.00, *p* = .003, *r* = .20). However, children in the 11-12y age group did not produce more mental state words than did children in the 9-10y age group (Mann-Whitney test: *Z* = 1.21, *p* = .225, *r* = .08).

Planned nonparametric post hoc analyses were conducted to follow up on the peer gender conformity x age group interaction. Results were consistent with parametric post hoc analyses. Children in the 5-6y age group did not differ in mental state word frequency proportion when describing GN peers and GC peers (Wilcoxon signed-rank test: *Z* = −0.75, *p* = .455, *r* = .07). Children in the 7-8y age group also did not differ in mental state word frequency proportion when describing GN peers and GC peers (Wilcoxon signed-rank test: *Z* = 1.36, *p* = .175, *r* = .13). Nevertheless, children in the 9-10y age group used fewer mental state words to describe GN peers than to describe GC peers (Wilcoxon signed rank test: *Z* = −2.21, *p* = .027, *r* = .21). Children in the 11-12y age group also used fewer mental state words to describe GN peers than to describe GC peers (Wilcoxon signed rank test: *Z* = −2.25, *p* = .024, *r* = .21).

1. **Nonparametric ANOVA table on diversity proportion of mental state words (*N* = 435)**

| **Variables** | ***F*** | ***p*** | ***η_p_^2^*** |
| --- | --- | --- | --- |
| Peer Gender | 2.55 | .111 | .01 |
| Peer Gender x Age Group | 2.24 | .083 | .02 |
| Peer Gender x Participant Gender | 0.34 | .557 | .00 |
| Peer Gender x Age Group x Participant Gender | 0.17 | .917 | .00 |
| Peer Gender Conformity | 3.57 | .059 | .01 |
| Peer Gender Conformity x Age Group | 4.39 | .005 | .03 |
| Peer Gender Conformity x Participant Gender | 1.76 | .186 | .00 |
| Peer Gender Conformity x Age Group x Participant Gender | 0.60 | .617 | .00 |
| Peer Gender x Peer Gender Conformity | 0.58 | .447 | .00 |
| Peer Gender x Peer Gender Conformity x Age Group | 1.72 | .162 | .01 |
| Peer Gender x Peer Gender Conformity x Participant Gender | 0.03 | .860 | .00 |
| Peer Gender x Peer Gender Conformity x Age Group x Participant Gender | 0.08 | .971 | .00 |
| Age Group | 24.24 | < .001 | .15 |
| Participant Gender | 0.32 | .573 | .00 |
| Age Group x Participant Gender | 0.18 | .910 | .00 |

Nonparametric results were highly similar to parametric results. Nonparametric analyses showed a significant main effect of age group, *F*(3, 427) = 24.24, *p* < .001, η_p_^2^ = .15, and a significant peer gender conformity x age group interaction, *F*(3, 427) = 4.39, *p* = .005, η_p_^2^ = .03. The main effect of peer gender conformity was marginally significant, *F*(1, 427) = 3.57, *p* = .059, η_p_^2^ =.01.

Regarding the main effect of age group, older children generally produced a higher diversity proportion of mental state words than did younger children when describing peers. Children in the 7-8y age group produced more diverse mental state words than did children in the 5-6y age group (Mann-Whitney test: *Z* = 2.60, *p* = .009, *r* = .18); children in the 9-10y age group produced more diverse mental state words than did children in the 7-8y age group (Mann-Whitney test: *Z* = 3.22, *p* = .001, *r* = .22). However, children in the 11-12y age group did not produce more diverse mental state words than did children in the 9-10y age group (Mann-Whitney test: *Z* = 1.02, *p* = .308, *r* = .07).

Planned nonparametric post hoc analyses were conducted to follow up on the peer gender conformity x age group interaction. Results were consistent with those from parametric post hoc analyses. Children in the 5-6y age group did not differ in mental state word diversity proportion when describing GN peers and GC peers (Wilcoxon signed-rank test: *Z* = −0.91, *p* = .365, *r* = .09). Children in the 7-8y age group also did not differ in mental state word diversity proportion when describing GN peers and GC peers (Wilcoxon signed-rank test: *Z* = 1.50, *p* = .133, *r* = .15). Nevertheless, children in the 9-10y age group used less diverse mental state words when describing GN peers than when describing GC peers (Wilcoxon signed-rank test: *Z* = −2.55, *p* = .011, *r* = .24). Children in the 11-12y age group also used less diverse mental state words when describing GN peers than when describing GC peers (Wilcoxon signed-rank test: *Z* = −2.80, *p* = .005, *r* = .27).

1. **Nonparametric ANOVA table on bullying tendency (*N* = 449)**

| **Variables** | ***F*** | ***p*** | ***η_p_^2^*** |
| --- | --- | --- | --- |
| Peer Gender | 1.54 | .215 | .00 |
| Peer Gender x Age Group | 2.11 | .099 | .01 |
| Peer Gender x Participant Gender | 3.57 | .060 | .01 |
| Peer Gender x Age Group x Participant Gender | 1.82 | .143 | .01 |
| Peer Gender Conformity | 55.57 | < .001 | .11 |
| Peer Gender Conformity x Age Group | 4.37 | .005 | .03 |
| Peer Gender Conformity x Participant Gender | 3.95 | .048 | .01 |
| Peer Gender Conformity x Age Group x Participant Gender | 1.72 | .161 | .01 |
| Peer Gender x Peer Gender Conformity | 3.23 | .073 | .01 |
| Peer Gender x Peer Gender Conformity x Age Group | 0.60 | .618 | .00 |
| Peer Gender x Peer Gender Conformity x Participant Gender | 6.00 | .015 | .01 |
| Peer Gender x Peer Gender Conformity x Age Group x Participant Gender | 1.87 | .133 | .01 |
| Age Group | 7.11 | < .001 | .05 |
| Participant Gender | 7.36 | .007 | .02 |
| Age Group x Participant Gender | 3.94 | .009 | .03 |

Consistent with the results of parametric ANOVA, the main effect of peer gender conformity, *F*(1, 441) = 55.57, *p* < .001, η_p_^2^ = .11, and the main effect of age group, *F*(3, 441) = 7.11, *p* < .001, η_p_^2^ = .05, were significant in the nonparametric ANOVA. Participants reported higher bullying tendency towards GN peers compared to GC peers, and participants in the 5-6y age group reported lower bullying tendency towards hypothetical peers than older age groups. The age group x participant gender interaction, *F*(3, 441) = 3.94, *p* = .009, η_p_^2^ = .03, and the peer gender x peer gender conformity x participant gender interaction, *F*(1, 441) = 6.00, *p* = .015, η_p_^2^ = .01, also remained significant in the nonparametric ANOVA.

Regarding the age group x participant gender interaction, similar to parametric analyses, boy participants reported higher bullying tendency towards hypothetical peers than girl participants only in the 5-6y age group (Mann-Whitney test: *Z* = 2.04, *p* = .041, *r* = .20), but not in the 7-8y (Mann-Whitney test: *Z* = −0.52, *p* = .603, *r* = .05), 9-10y (Mann-Whitney test: *Z* = 0.38, *p* = .704, *r* = .04), or 11-12y age group (Mann-Whitney test: *Z* = −0.54, *p* = .592, *r* = .05).

Regarding the peer gender x peer gender conformity x participant gender interaction, nonparametric post hoc analyses yielded results consistent with those from parametric post hoc analyses: boy participants reported lower bullying tendency to GC boys than to GC girls (Wilcoxon signed-rank test: *Z* = −2.91, *p* = .004, *r* = .20), while girl participants reported similar bullying tendency towards GC boys and girls (Wilcoxon signed-rank test: *Z* = 0.98, *p* = .326, *r* = .07); both boy participants (Wilcoxon signed-rank test: *Z* = 1.30, *p* = .194, *r* = .09) and girl participants (Wilcoxon signed-rank test: *Z* = −0.06, *p* = .955, *r* = .00), reported similar bullying tendency towards GN boys and girls.

In addition, the main effect of participant gender, *F*(1, 441) = 7.36, *p* = .007, η_p_^2^ = .02, peer gender conformity x age group interaction, *F*(3, 441) = 4.37, *p* = .005, η_p_^2^ = .03, and the peer gender conformity x participant gender interaction, *F*(1, 441) = 3.95, *p* = .048, η_p_^2^ = .01, were significant in the nonparametric ANOVA. Regarding the main effect of participant gender, boy participants reported higher bullying tendency towards hypothetical peers than did girl participants. Post hoc analyses were conducted to investigate the peer gender conformity x age group and peer gender conformity x participant gender interactions. Participants reported significantly higher bullying tendency towards GN peers than GC peers in the 5-6y age group (Wilcoxon signed-rank test: *Z* = 2.70, *p* = .007, *r* = .26), 7-8y age group (Wilcoxon signed-rank test: *Z* = 2.12 *p* = .034, *r* = .20), 9-10y age group (Wilcoxon signed-rank test: *Z* = 5.26, *p* < .001, *r* = .49), and 11-12y age groups (Wilcoxon signed-rank test: *Z* = 5.20, *p* < .001, *r* = .48). Although the magnitudes of differences in the age groups varied, participants in all age groups consistently reported higher bullying tendency towards GN peers than GC peers. Although there was a weak significant interaction between peer gender conformity and participant gender, both boy participants (Wilcoxon signed rank test: *Z* = 5.50, *p* < .001, *r* = .37) and girl participants (Wilcoxon signed rank test: *Z* = 5.31, *p* < .001, *r* = .35) reported higher bullying tendency towards GN peers than GC peers; boy and girl participants did not report bullying tendency towards GC peers (Mann-Whitney test: *Z* = 0.98, *p* = .329, *r* = .05) and GN peers (Mann-Whitney test: *Z* = 0.67, *p* = .502, *r* = .03) differently.

1. **Testing of moderated mediation models**
   1. **Model 1: Mediation via blatant humanness rating (*N* = 432)**

| Predictor variables | *b* | *SE* | *t* | *p* | 95% CI |
| --- | --- | --- | --- | --- | --- |
| Mediator model (DV: Blatant humanness rating) | | | | | |
| Peer gender conformity (path a) | 1.053 | 2.658 | 0.396 | .692 | −4.171, 6.278 |
| Age group | 0.642 | 0.666 | 0.963 | .336 | −0.668, 1.951 |
| Peer gender conformity x age group | −4.033 | 0.947 | −4.261 | < .001 | −5.894, −2.173 |
| DV model (DV: Bullying tendency) | | | | | |
| Peer gender conformity (path c’) | 0.140 | 0.026 | 5.490 | < .001 | 0.090, 0.190 |
| Blatant humanness rating (path b) | −0.005 | 0.001 | −4.820 | < .001 | −0.007, −0.003 |
|  | Estimate | | 95% CI | | |
| Index of Moderated Mediation | .021 | | **.009, .035** | | |

**Remarks**. Peer gender conformity (0 = gender conforming; 1 = gender nonconforming); *b* = unstandardized regression coefficients, *SE* = standard error; CI = confidence interval; Bootstrap sample size = 10,000.

- 1. **Model 2: Mediation via frequency proportion of mental state words (*N* = 427)**

| Predictor variables | *b* | *SE* | *t* | *p* | 95% CI |
| --- | --- | --- | --- | --- | --- |
| Mediator model (DV: Frequency proportion) | | | | | |
| Peer gender conformity (path a) | 0.003 | 0.003 | 1.010 | .313 | −0.002, 0.007 |
| Age group | 0.005 | 0.001 | 7.053 | < .001 | 0.004, 0.007 |
| Peer gender conformity x age group | −0.002 | 0.001 | −2.019 | .044 | −0.004, 0.000 |
| DV model (DV: Bullying tendency) | | | | | |
| Peer gender conformity (path c’) | 0.203 | 0.026 | 7.938 | < .001 | 0.153, 0.254 |
| Frequency proportion (path b) | 1.954 | 1.230 | 1.588 | .113 | −0.464, 4.372 |
|  | Estimate | | 95% CI | | |
| Index of Moderated Mediation | −.004 | | −.011, .001 | | |

**Remarks**. Peer gender conformity (0 = gender conforming; 1 = gender nonconforming); *b* = unstandardized regression coefficients, *SE* = standard error; CI = confidence interval; Bootstrap sample size = 10,000.

- 1. **Model 3: Mediation via diversity proportion of mental state words (*N* = 423)**

| Predictor variables | *b* | *SE* | *t* | *p* | 95% CI |
| --- | --- | --- | --- | --- | --- |
| Mediator model (DV: Diversity proportion) | | | | | |
| Peer gender conformity (path a) | 0.003 | 0.002 | 1.559 | .120 | −0.001, 0.006 |
| Age group | 0.003 | 0.001 | 6.653 | < .001 | 0.002, 0.004 |
| Peer gender conformity x age group | −0.002 | 0.001 | −2.837 | .005 | −0.003, −0.001 |
| DV model (DV: Bullying tendency) | | | | | |
| Peer gender conformity (path c’) | 0.192 | 0.026 | 7.419 | < .001 | 0.141, 0.243 |
| Diversity proportion (path b) | 1.738 | 1.936 | 0.898 | .370 | −2.067, 5.544 |
|  | Estimate | | 95% CI | | |
| Index of Moderated Mediation | −.003 | | −.011, .003 | | |

**Remarks**. Peer gender conformity (0 = gender conforming; 1 = gender nonconforming); *b* = unstandardized regression coefficients, *SE* = standard error; CI = confidence interval; Bootstrap sample size = 10,000.

1. **Testing of mediation models by age group**
   1. **Model 1: Mediation via blatant humanness rating**

| Predictor variables | *b* | *SE* | *t* | *p* | 95% CI |
| --- | --- | --- | --- | --- | --- |
| **5-6y age group (*N* = 99)** | | | | | |
| Mediator model (DV: Blatant humanness rating) | | | | | |
| Peer gender conformity (path a) | −0.828 | 2.040 | −0.406 | .686 | −4.877, 3.220 |
| DV model (DV: Bullying tendency) | | | | | |
| Peer gender conformity (path c’) | 0.157 | 0.058 | 2.731 | .008 | 0.043, 0.272 |
| Blatant humanness rating (path b) | −0.003 | 0.003 | −1.150 | .253 | −0.009, 0.002 |
| **7-8y age group (*N* = 103)** |  |  |  |  |  |
| Mediator model (DV: Blatant humanness rating) | | | | | |
| Peer gender conformity (path a) | −9.500 | 2.343 | −4.054 | < .001 | −14.148, −4.852 |
| DV model (DV: Bullying tendency) |  |  |  |  |  |
| Peer gender conformity (path c’) | 0.074 | 0.051 | 1.440 | .153 | −0.028, 0.176 |
| Blatant humanness rating (path b) | −0.004 | 0.002 | −1.716 | .089 | −0.008, 0.001 |
| **9-10y age group (*N* = 112)** |  |  |  |  |  |
| Mediator model (DV: Blatant humanness rating) | | | | | |
| Peer gender conformity (path a) | −12.179 | 2.060 | −5.911 | < .001 | −16.261, −8.096 |
| DV model (DV: Bullying tendency) |  |  |  |  |  |
| Peer gender conformity (path c’) | 0.158 | 0.044 | 3.571 | < .001 | 0.070, 0.246 |
| Blatant humanness rating (path b) | −0.005 | 0.002 | −2.804 | .006 | −0.009, −0.002 |
| **11-12y age group (*N* = 118)** |  |  |  |  |  |
| Mediator model (DV: Blatant humanness rating) | | | | | |
| Peer gender conformity (path a) | −13.640 | 2.005 | −6.803 | < .001 | −17.611, −9.669 |
| DV model (DV: Bullying tendency) |  |  |  |  |  |
| Peer gender conformity (path c’) | 0.143 | 0.053 | 2.700 | .008 | 0.038, 0.249 |
| Blatant humanness rating (path b) | −0.008 | 0.002 | −3.820 | < .001 | −0.012, −0.004 |
| Indirect effects | *b* | *SE* | *Z* | *p* | 95% CI |
| 5-6y age group | 0.003 | 0.009 | 0.296 | .767 | −0.015, 0.025 |
| 7-8y age group | 0.033 | 0.021 | 1.541 | .123 | −0.005, 0.081 |
| 9-10y age group | 0.061 | 0.024 | 2.504 | **.012** | **0.017, 0.114** |
| 11-12y age group | 0.108 | 0.033 | 3.304 | **.001** | **0.048, 0.180** |

**Remarks**. Peer gender conformity (0 = gender conforming; 1 = gender nonconforming); *b* = unstandardized regression coefficients, *SE* = standard error; CI = confidence interval; DV = dependent variable; Bootstrap sample size = 10,000.

- 1. **Model 2: Mediation via frequency proportion of mental state words**

| Predictor variables | *b* | *SE* | *t* | *p* | 95% CI |
| --- | --- | --- | --- | --- | --- |
| **5-6y age group (*N* = 100)** | | | | | |
| Mediator model (DV: Frequency Proportion) | | | | | |
| Peer gender conformity (path a) | −0.001 | 0.002 | −0.366 | .715 | −0.005, 0.003 |
| DV model (DV: Bullying tendency) | | | | | |
| Peer gender conformity (path c’) | 0.181 | 0.058 | 3.135 | .002 | 0.066, 0.295 |
| Frequency Proportion (path b) | 7.952 | 2.873 | 2.768 | .007 | 2.251, 13.653 |
| **7-8y age group (*N* = 105)** |  |  |  |  |  |
| Mediator model (DV: Frequency Proportion) | | | | | |
| Peer gender conformity (path a) | 0.002 | 0.002 | 1.069 | .288 | −0.002, 0.006 |
| DV model (DV: Bullying tendency) |  |  |  |  |  |
| Peer gender conformity (path c’) | 0.127 | 0.056 | 2.263 | .026 | 0.016, 0.239 |
| Frequency Proportion (path b) | 2.757 | 2.681 | 1.028 | .306 | −2.560, 8.074 |
| **9-10y age group (*N* = 109)** |  |  |  |  |  |
| Mediator model (DV: Frequency Proportion) | | | | | |
| Peer gender conformity (path a) | −0.005 | 0.002 | −2.530 | .013 | −0.009, −0.001 |
| DV model (DV: Bullying tendency) |  |  |  |  |  |
| Peer gender conformity (path c’) | 0.228 | 0.042 | 5.441 | < .001 | 0.145, 0.311 |
| Frequency Proportion (path b) | 1.533 | 1.890 | 0.811 | .419 | −2.213, 5.279 |
| **11-12y age group (*N* = 113)** |  |  |  |  |  |
| Mediator model (DV: Frequency Proportion) | | | | | |
| Peer gender conformity (path a) | −0.004 | 0.002 | −2.304 | .023 | −0.008, −0.001 |
| DV model (DV: Bullying tendency) |  |  |  |  |  |
| Peer gender conformity (path c’) | 0.250 | 0.049 | 5.146 | < .001 | 0.154, 0.346 |
| Frequency Proportion (path b) | −2.625 | 2.446 | −1.073 | .286 | −7.472, 2.222 |
| Indirect effects | *b* | *SE* | *Z* | *p* | 95% CI |
| 5-6y age group | −0.006 | 0.017 | −0.342 | .733 | −0.043, 0.027 |
| 7-8y age group | 0.006 | 0.010 | 0.614 | .539 | −0.009, 0.029 |
| 9-10y age group | −0.008 | 0.011 | −0.723 | .470 | −0.033, 0.012 |
| 11-12y age group | 0.011 | 0.012 | 0.905 | .365 | −0.010, 0.040 |

**Remarks**. Peer gender conformity (0 = gender conforming; 1 = gender nonconforming); *b* = unstandardized regression coefficients, *SE* = standard error; CI = confidence interval; DV = dependent variable; Bootstrap sample size = 10,000.

- 1. **Model 3: Mediation via diversity proportion of mental state words**

| Predictor variables | *b* | *SE* | *t* | *p* | 95% CI |
| --- | --- | --- | --- | --- | --- |
| **5-6y age group (*N* = 102)** | | | | | |
| Mediator model (DV: Diversity Proportion) | | | | | |
| Peer gender conformity (path a) | 0.000 | 0.001 | −0.149 | .882 | −0.003, 0.003 |
| DV model (DV: Bullying tendency) | | | | | |
| Peer gender conformity (path c’) | 0.164 | 0.057 | 2.883 | .005 | 0.051, 0.277 |
| Diversity Proportion (path b) | 10.742 | 4.226 | 2.542 | .013 | 2.357, 19.127 |
| **7-8y age group (*N* = 100)** |  |  |  |  |  |
| Mediator model (DV: Diversity Proportion) | | | | | |
| Peer gender conformity (path a) | 0.002 | 0.001 | 1.353 | .179 | −0.001, 0.004 |
| DV model (DV: Bullying tendency) |  |  |  |  |  |
| Peer gender conformity (path c’) | 0.122 | 0.059 | 2.073 | .041 | 0.005, 0.239 |
| Diversity Proportion (path b) | 1.704 | 4.470 | 0.381 | .704 | −7.166, 10.574 |
| **9-10y age group (*N* = 110)** |  |  |  |  |  |
| Mediator model (DV: Diversity Proportion) | | | | | |
| Peer gender conformity (path a) | −0.004 | 0.001 | −3.088 | .003 | −0.007, −0.002 |
| DV model (DV: Bullying tendency) |  |  |  |  |  |
| Peer gender conformity (path c’) | 0.227 | 0.042 | 5.356 | < .001 | 0.143, 0.311 |
| Diversity Proportion (path b) | 0.950 | 2.946 | 0.323 | .748 | −4.890, 6.790 |
| **11-12y age group (*N* = 111)** |  |  |  |  |  |
| Mediator model (DV: Diversity Proportion) | | | | | |
| Peer gender conformity (path a) | −0.004 | 0.001 | −3.213 | .002 | −0.006, −0.001 |
| DV model (DV: Bullying tendency) |  |  |  |  |  |
| Peer gender conformity (path c’) | 0.224 | 0.051 | 4.378 | < .001 | 0.123, 0.325 |
| Diversity Proportion (path b) | −4.436 | 4.083 | −1.086 | .280 | −12.529, 3.657 |
| Indirect effects | *b* | *SE* | *Z* | *p* | 95% CI |
| 5-6y age group | −0.002 | 0.016 | −0.138 | .890 | −0.035, 0.028 |
| 7-8y age group | 0.003 | 0.010 | 0.299 | .765 | −0.016, 0.026 |
| 9-10y age group | −0.004 | 0.013 | −0.305 | .760 | −0.031, 0.022 |
| 11-12y age group | 0.016 | 0.017 | 0.987 | .324 | −0.014, 0.054 |

**Remarks**. Peer gender conformity (0 = gender conforming; 1 = gender nonconforming); *b* = unstandardized regression coefficients, *SE* = standard error; CI = confidence interval; DV = dependent variable; Bootstrap sample size = 10,000.

1. **Testing of reverse mediation models by age group**
   1. **Model 1: Reverse mediation with blatant humanness rating**

| Predictor variables | *b* | *SE* | *t* | *p* | 95% CI |
| --- | --- | --- | --- | --- | --- |
| **5-6y age group (*N* = 99)** | | | | | |
| Mediator model (DV: Bullying tendency) | | | | | |
| Peer gender conformity (path a) | 0.160 | 0.058 | 2.775 | .007 | 0.046, 0.274 |
| DV model (DV: Blatant humanness rating) | | | | | |
| Peer gender conformity (path c’) | −0.172 | 2.115 | −0.081 | .936 | −4.370, 4.027 |
| Bullying tendency (path b) | −4.107 | 3.570 | −1.150 | .253 | −11.193, 2.980 |
| **7-8y age group (*N* = 103)** |  |  |  |  |  |
| Mediator model (DV: Bullying tendency) | | | | | |
| Peer gender conformity (path a) | 0.107 | 0.048 | 2.220 | .029 | 0.011, 0.202 |
| DV model (DV: Blatant humanness rating) | | | | | |
| Peer gender conformity (path c’) | −8.625 | 2.377 | −3.629 | < .001 | −13.340, −3.910 |
| Bullying tendency (path b) | −8.195 | 4.777 | −1.716 | .089 | −17.670, 1.281 |
| **9-10y age group (*N* = 112)** |  |  |  |  |  |
| Mediator model (DV: Bullying tendency) | | | | | |
| Peer gender conformity (path a) | 0.219 | 0.040 | 5.500 | < .001 | 0.140, 0.298 |
| DV model (DV: Blatant humanness rating) | | | | | |
| Peer gender conformity (path c’) | −9.252 | 2.256 | −4.102 | < .001 | −13.722, −4.782 |
| Bullying tendency (path b) | −13.378 | 4.772 | −2.804 | .006 | −22.834, −3.922 |
| **11-12y age group (*N* = 118)** |  |  |  |  |  |
| Mediator model (DV: Bullying tendency) | | | | | |
| Peer gender conformity (path a) | 0.251 | 0.048 | 5.293 | < .001 | 0.157, 0.346 |
| DV model (DV: Blatant humanness rating) | | | | | |
| Peer gender conformity (path c’) | −10.093 | 2.113 | −4.777 | < .001 | −14.278, −5.908 |
| Bullying tendency (path b) | −14.109 | 3.694 | −3.820 | < .001 | −21.425, −6.793 |
| Indirect effects | *b* | *SE* | *Z* | *p* | 95% CI |
| 5-6y age group | −0.657 | 0.652 | −1.008 | .313 | −2.106, 0.438 |
| 7-8y age group | −0.875 | 0.685 | −1.279 | .201 | −2.463, 0.136 |
| 9-10y age group | −2.926 | 1.187 | −2.466 | **.014** | −**5.462,** −**0.842** |
| 11-12y age group | −3.547 | 1.159 | −3.062 | **.002** | −**6.029,** −**1.544** |

**Remarks**. Peer gender conformity (0 = gender conforming; 1 = gender nonconforming); *b* = unstandardized regression coefficients, *SE* = standard error; CI = confidence interval; DV = dependent variable; Bootstrap sample size = 10,000.

Compared to the hypothesized mediation models (peer gender conformity → blatant humanness rating → bullying tendency), the reverse mediation models (peer gender conformity → bullying tendency → blatant humanness rating) generated indirect effects of larger *p* values in the 9-10y and 11-12y age groups. Thus, the hypothesized mediation models were retained.

- 1. **Model 2: Reverse mediation with frequency proportion of mental state words**

| Predictor variables | *b* | *SE* | *t* | *p* | 95% CI |
| --- | --- | --- | --- | --- | --- |
| **5-6y age group (*N* = 100)** | | | | | |
| Mediator model (DV: Bullying tendency) | | | | | |
| Peer gender conformity (path a) | 0.175 | 0.060 | 2.938 | .004 | 0.057, 0.293 |
| DV model (DV: Frequency proportion) | | | | | |
| Peer gender conformity (path c’) | −0.002 | 0.002 | −1.147 | .254 | −0.006, 0.002 |
| Bullying tendency (path b) | 0.009 | 0.003 | 2.768 | .007 | 0.003, 0.016 |
| **7-8y age group (*N* = 105)** |  |  |  |  |  |
| Mediator model (DV: Bullying tendency) | | | | | |
| Peer gender conformity (path a) | 0.133 | 0.056 | 2.383 | .019 | 0.022, 0.244 |
| DV model (DV: Frequency proportion) | | | | | |
| Peer gender conformity (path c’) | 0.002 | 0.002 | 0.807 | .422 | −0.003, 0.006 |
| Bullying tendency (path b) | 0.004 | 0.004 | 1.028 | .306 | −0.003, 0.011 |
| **9-10y age group (*N* = 109)** |  |  |  |  |  |
| Mediator model (DV: Bullying tendency) | | | | | |
| Peer gender conformity (path a) | 0.220 | 0.041 | 5.411 | < .001 | 0.140, 0.301 |
| DV model (DV: Frequency proportion) | | | | | |
| Peer gender conformity (path c’) | −0.006 | 0.002 | −2.615 | .010 | −0.011, −0.002 |
| Bullying tendency (path b) | 0.004 | 0.005 | 0.811 | .419 | −0.006, 0.014 |
| **11-12y age group (*N* = 113)** |  |  |  |  |  |
| Mediator model (DV: Bullying tendency) | | | | | |
| Peer gender conformity (path a) | 0.261 | 0.048 | 5.497 | < .001 | 0.167, 0.355 |
| DV model (DV: Frequency proportion) | | | | | |
| Peer gender conformity (path c’) | −0.003 | 0.002 | −1.552 | .124 | −0.007, 0.001 |
| Bullying tendency (path b) | −0.004 | 0.004 | −1.073 | .286 | −0.011, 0.003 |
| Indirect effects | *b* | *SE* | *Z* | *p* | 95% CI |
| 5-6y age group | 0.002 | 0.001 | 1.956 | .051 | 0.000, 0.003 |
| 7-8y age group | 0.001 | 0.001 | 0.881 | .378 | −0.001, 0.002 |
| 9-10y age group | 0.001 | 0.001 | 0.789 | .430 | −0.001, 0.003 |
| 11-12y age group | −0.001 | 0.001 | −1.037 | .300 | −0.003, 0.001 |

**Remarks**. Peer gender conformity (0 = gender conforming; 1 = gender nonconforming); *b* = unstandardized regression coefficients, *SE* = standard error; CI = confidence interval; DV = dependent variable; Bootstrap sample size = 10,000.

- 1. **Model 3: Reverse mediation with diversity proportion of mental state words**

| Predictor variables | *b* | *SE* | *t* | *p* | 95% CI |
| --- | --- | --- | --- | --- | --- |
| **5-6y age group (*N* = 102)** | | | | | |
| Mediator model (DV: Bullying tendency) | | | | | |
| Peer gender conformity (path a) | 0.162 | 0.058 | 2.772 | .007 | 0.046, 0.278 |
| DV model (DV: Diversity proportion) | | | | | |
| Peer gender conformity (path c’) | −0.001 | 0.001 | −0.823 | .413 | −0.004, 0.002 |
| Bullying tendency (path b) | 0.006 | 0.002 | 2.542 | .013 | 0.001, 0.010 |
| **7-8y age group (*N* = 100)** |  |  |  |  |  |
| Mediator model (DV: Bullying tendency) | | | | | |
| Peer gender conformity (path a) | 0.125 | 0.058 | 2.154 | .034 | 0.010, 0.240 |
| DV model (DV: Diversity proportion) | | | | | |
| Peer gender conformity (path c’) | 0.002 | 0.001 | 1.236 | .220 | −0.001, 0.004 |
| Bullying tendency (path b) | 0.001 | 0.002 | 0.381 | .704 | −0.004, 0.005 |
| **9-10y age group (*N* = 110)** |  |  |  |  |  |
| Mediator model (DV: Bullying tendency) | | | | | |
| Peer gender conformity (path a) | 0.223 | 0.040 | 5.513 | < .001 | 0.143, 0.303 |
| DV model (DV: Diversity proportion) | | | | | |
| Peer gender conformity (path c’) | −0.004 | 0.002 | −2.870 | .005 | −0.007, −0.001 |
| Bullying tendency (path b) | 0.001 | 0.003 | 0.323 | .748 | −0.005, 0.007 |
| **11-12y age group (*N* = 111)** |  |  |  |  |  |
| Mediator model (DV: Bullying tendency) | | | | | |
| Peer gender conformity (path a) | 0.240 | 0.049 | 4.908 | < .001 | 0.143, 0.337 |
| DV model (DV: Diversity proportion) | | | | | |
| Peer gender conformity (path c’) | −0.003 | 0.001 | −2.452 | .016 | −0.006, −0.001 |
| Bullying tendency (path b) | −0.002 | 0.002 | −1.086 | .280 | −0.007, 0.002 |
| Indirect effects | *b* | *SE* | *Z* | *p* | 95% CI |
| 5-6y age group | 0.001 | 0.001 | 1.811 | .070 | 0.000, 0.002 |
| 7-8y age group | 0.000 | 0.000 | 0.342 | .733 | −0.001, 0.001 |
| 9-10y age group | 0.000 | 0.001 | 0.317 | .751 | −0.001, 0.002 |
| 11-12y age group | −0.001 | 0.001 | −1.040 | .298 | −0.002, 0.001 |

**Remarks**. Peer gender conformity (0 = gender conforming; 1 = gender nonconforming); *b* = unstandardized regression coefficients, *SE* = standard error; CI = confidence interval; DV = dependent variable; Bootstrap sample size = 10,000.

1. **Parametric analyses based on winsorized data**

Identical parametric analyses using winsorized data were conducted. In these parametric analyses, outliers (z ≥ 3.29 or ≤ -3.29) were winsorized to the nearest non-outlier values instead of being excluded from analyses. Winsorization is a less conservative approach that allows a bigger sample size and allows participants with extreme values to contribute to the statistical findings.

Analyses based on winsorized data yielded results highly similar to the original analyses based on data after outlier exclusion. After winsorization, the main effect of peer gender conformity was significant for blatant humanness rating, *F*(1, 455) = 69.34, *p* < .001, η_p_^2^ = .13, frequency proportion measure, *F*(1, 449) = 4.07, *p* = .044, η_p_^2^ = .01, diversity proportion measure, *F*(1, 449) = 4.54, *p* = .034, η_p_^2^ = .01, and bullying tendency, *F*(1, 455) = 54.48, *p* < .001, η_p_^2^ = .11.

The peer gender conformity x age group interaction also remained significant for blatant humanness rating, *F*(3, 455) = 7.78, *p* < .001, η_p_^2^ = .05, and the diversity proportion measure, *F*(3, 449) = 3.43, *p* = .017, η_p_^2^ = .02. For the frequency proportion measure, the peer gender conformity x age group interaction was marginally significant, *F*(3, 449) = 2.27, *p* = .079, η_p_^2^ = .02. Post hoc tests based on winsorized data revealed the same age pattern as the original analyses: children rated GN peers as significantly less human-like/ more insect-like than GC peers in the 7-8y, 9-10y, and 11-12y age groups, but not in the 5-6y age group; children used significantly fewer and less diverse mental state words to describe GN than GC peers in the 9-10y and 11-12y age groups, but not in the 5-6y or 7-8y age group.

The peer gender x peer gender conformity x participant gender interaction also remained significant for bullying tendency, *F*(1, 455) = 7.68, *p* = .006, η_p_^2^ = .02. Post hoc analyses yielded patterns consistent with those from the original analyses. For GC peers, boy participants reported a significantly lower bullying tendency toward GC boys than GC girls (*p* = .005); girl participants did not report significantly different bullying tendency towards GC boys and GC girls. For GN peers, both boy and girl participants did not rate GN peers differently based on their gender and peers’ gender.

Multilevel moderated mediation analyses based on winsorized data also yielded patterns similar to the original analyses. The index of moderated mediation was significant only when blatant humanness rating was the mediator of the model (estimate = .019, 95% CI [.008, .032]), but not when either frequency proportion (estimate = −.002, 95% CI [−.007, .003]) or diversity proportion (estimate = −.002, 95% CI [−.008, .003]) was included as the mediator. When testing the blatant humanness mediation model (i.e., peer gender conformity → blatant humanness rating → bullying tendency) for each age group separately, similar to the original analyses, the indirect effects were non-significant in the 5-6y age group (*b* = 0.00, *SE* = 0.01, *Z* = 0.05, *p* = .964, 95% CI [−.012, .013]) and in the 7-8y age group (*b* = 0.03, *SE* = 0.02, *Z* = 1.60, *p* = .109, 95% CI [−.002, .080]), but were significant in the 9-10y age group (*b* = 0.06, *SE* = 0.03, *Z* = 2.11, *p* = .035, 95% CI [.007, .117]) and in the 11-12y age group (*b* = 0.11, *SE* = 0.03, *Z* = 3.30, *p* = .001, 95% CI [.048, .180]). When frequency and diversity proportion measures were included as mediators, similar to the original analyses, the indirect effects were non-significant for all age groups.

1. **Testing of multilevel parallel moderated mediation model with multiple humanness mediators**
2. **Conceptual model of the multilevel parallel moderated mediation model**

Following a peer review suggestion, a moderated mediation model including all three humanness measures as mediators simultaneously was tested.


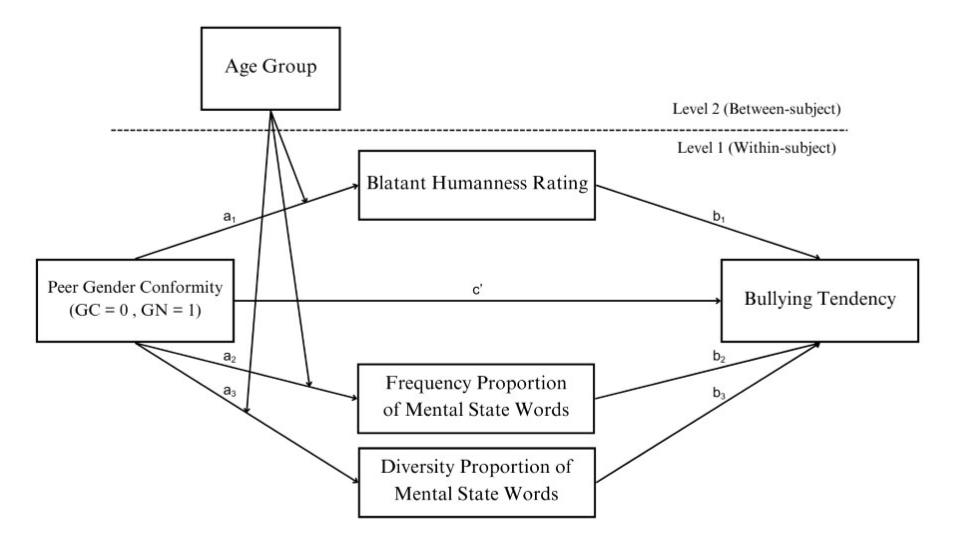


The latest version of the MLmed SPSS macro used in the original analyses only allows moderation analyses for paths involving the first mediator in a parallel mediation model. Due to this limitation, separate parallel mediation analyses by each age group were conducted.

1. **Testing of multilevel parallel mediation models by age group**

| Predictor variables | *b* | *SE* | *t* | *p* | 95% CI |
| --- | --- | --- | --- | --- | --- |
| **5-6y age group (*N* = 91)** | | | | | |
| Mediator model (DV: Blatant humanness rating) | | | | | |
| Peer gender conformity (path a_1_) | −0.346 | 2.109 | −0.164 | .870 | −4.537, 3.845 |
| Mediator model (DV: Frequency proportion) | | | |  |  |
| Peer gender conformity (path a_2_) | −0.002 | 0.002 | −1.047 | .298 | −0.006, 0.002 |
| Mediator model (DV: Diversity proportion) | | | |  |  |
| Peer gender conformity (path a_3_) | −0.001 | 0.001 | −0.987 | .327 | −0.004, 0.001 |
| DV model (DV: Bullying tendency) | | | | | |
| Peer gender conformity (path c’) | 0.180 | 0.060 | 3.007 | .004 | 0.061, 0.298 |
| Blatant humanness rating (path b_1_) | −0.005 | 0.003 | −1.779 | .079 | −0.011, 0.001 |
| Frequency proportion (path b_2_) | 2.716 | 6.233 | 0.436 | .664 | −9.673, 15.105 |
| Diversity proportion (path b_3_) | 11.277 | 10.225 | 1.103 | .273 | −9.047, 31.601 |
| **7-8y age group (*N* = 93)** |  |  |  |  |  |
| Mediator model (DV: Blatant humanness rating) | | | | | |
| Peer gender conformity (path a_1_) | −8.925 | 2.473 | −3.608 | < .001 | −13.837, −4.013 |
| Mediator model (DV: Frequency proportion) | | | | | |
| Peer gender conformity (path a_2_) | 0.003 | 0.002 | 1.378 | .172 | −0.001, 0.007 |
| Mediator model (DV: Diversity proportion) | |  |  |  |  |
| Peer gender conformity (path a_3_) | 0.002 | 0.001 | 1.513 | .134 | −0.001, 0.005 |
| DV model (DV: Bullying tendency) |  |  |  |  |  |
| Peer gender conformity (path c’) | 0.068 | 0.056 | 1.217 | .227 | −0.043, 0.179 |
| Blatant humanness rating (path b_1_) | −0.004 | 0.002 | −1.925 | .058 | −0.009, 0.000 |
| Frequency proportion (path b_2_) | 3.798 | 4.770 | 0.796 | .428 | −5.680, 13.277 |
| Diversity proportion (path b_3_) | −4.078 | 7.203 | −0.566 | .573 | −18.390, 10.235 |
| **9-10y age group (*N* = 112)** |  |  |  |  |  |
| Mediator model (DV: Blatant humanness rating) | | | | | |
| Peer gender conformity (path a_1_) | −12.179 | 2.060 | −5.911 | < .001 | −16.261, −8.096 |
| Mediator model (DV: Frequency proportion) | | |  |  |  |
| Peer gender conformity (path a_2_) | −0.005 | 0.002 | −2.530 | .013 | −0.009, −0.001 |
| Mediator model (DV: Diversity proportion) | |  |  |  |  |
| Peer gender conformity (path a_3_) | −0.004 | 0.001 | −2.900 | .005 | −0.006, −0.001 |
| DV model (DV: Bullying tendency) |  |  |  |  |  |
| Peer gender conformity (path c’) | 0.170 | 0.047 | 3.648 | < .001 | 0.077, 0.262 |
| Blatant humanness rating (path b_1_) | −0.005 | 0.002 | −2.695 | .008 | −0.009, −0.001 |
| Frequency proportion (path b_2_) | 1.855 | 3.206 | 0.579 | .564 | −4.502, 8.212 |
| Diversity proportion (path b_3_) | −0.396 | 5.198 | −0.076 | .939 | −10.702, 9.910 |
| **11-12y age group (*N* = 113)** |  |  |  |  |  |
| Mediator model (DV: Blatant humanness rating) | | | | | |
| Peer gender conformity (path a_1_) | −14.204 | 2.011 | −7.062 | < .001 | −18.188, -10.219 |
| Mediator model (DV: Frequency proportion) | |  |  |  |  |
| Peer gender conformity (path a_2_) | −0.005 | 0.002 | −2.768 | .007 | −0.009, −0.001 |
| Mediator model (DV: Diversity proportion) | |  |  |  |  |
| Peer gender conformity (path a_3_) | −0.004 | 0.001 | −3.337 | .001 | −0.006, −0.002 |
| DV model (DV: Bullying tendency) |  |  |  |  |  |
| Peer gender conformity (path c’) | 0.132 | 0.057 | 2.298 | .024 | 0.018, 0.246 |
| Blatant humanness rating (path b_1_) | −0.007 | 0.002 | −3.407 | < .001 | −0.012, −0.003 |
| Frequency proportion (path b_2_) | −3.828 | 4.151 | −0.922 | .359 | −12.058, 4.403 |
| Diversity proportion (path b_3_) | 0.621 | 6.609 | 0.094 | .925 | −12.482, 13.725 |
| Indirect effects | *b* | *SE* | *Z* | *p* | 95% CI |
| **5-6y age group** |  |  |  |  |  |
| Blatant humanness rating (a_1_b_1_) | 0.002 | 0.013 | 0.143 | .887 | −0.026, 0.031 |
| Frequency proportion (a_2_b_2_) | −0.006 | 0.020 | −0.302 | .763 | −0.054, 0.031 |
| Diversity proportion (a_3_b_3_) | −0.014 | 0.024 | −0.609 | .542 | −0.072, 0.023 |
| **7-8y age group** |  |  |  |  |  |
| Blatant humanness rating (a_1_b_1_) | 0.037 | 0.023 | 1.650 | .099 | −0.001, 0.087 |
| Frequency proportion (a_2_b_2_) | 0.011 | 0.019 | 0.584 | .559 | −0.020, 0.056 |
| Diversity proportion (a_3_b_3_) | −0.008 | 0.019 | −0.451 | .652 | −0.053, 0.026 |
| **9-10y age group** |  |  |  |  |  |
| Blatant humanness rating (a_1_b_1_) | 0.060 | 0.025 | 2.423 | **.015** | **0.015, 0.112** |
| Frequency proportion (a_2_b_2_) | −0.010 | 0.019 | −0.526 | .599 | −0.051, 0.025 |
| Diversity proportion (a_3_b_3_) | 0.002 | 0.020 | 0.072 | .943 | −0.040, 0.045 |
| **11-12y age group** |  |  |  |  |  |
| Blatant humanness rating (a_1_b_1_) | 0.105 | 0.034 | 3.044 | **.002** | **0.042, 0.176** |
| Frequency proportion (a_2_b_2_) | 0.019 | 0.023 | 0.828 | .408 | −0.023, 0.071 |
| Diversity proportion (a_3_b_3_) | −0.002 | 0.026 | −0.090 | .928 | −0.057, 0.051 |

**Remarks**. Peer gender conformity (0 = gender conforming; 1 = gender nonconforming); *b* = unstandardized regression coefficients, *SE* = standard error; CI = confidence interval; DV = dependent variable; Bootstrap sample size = 10,000.

Consistent with the original models, significant indirect effects via blatant humanness ratings were observed only in the 9-10y and 11-12y age groups. By contrast, indirect effects mediated by frequency proportion and diversity proportion were not significant in any age group.

1. **Testing of multilevel moderated mediation models including participant gender and peer gender as moderators**
2. **Conceptual model of the multilevel moderated mediation model**

Given the significant peer gender x peer gender conformity x participant gender interaction in predicting bullying tendency, in response to a peer review suggestion, an additional model was tested. This model included participant gender and peer gender as moderators of the path between gender conformity and bullying tendency in the original model.


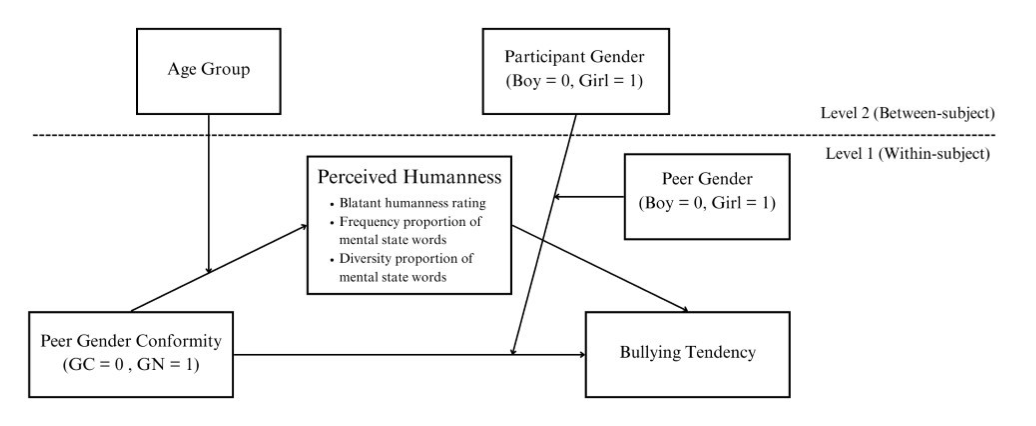


The latest version of the MLmed SPSS macro used in our original analyses only allows one level-2 variable to serve as a moderator for each path. Due to this limitation, separate moderated mediation analyses were conducted for each peer gender condition (i.e., boy and girl).

1. **Testing of multilevel moderated mediation models for boy peer condition**

**Model 1: Mediation via blatant humanness rating (*N* = 432)**

| Predictor variables | *b* | *SE* | *t* | *p* | 95% CI |
| --- | --- | --- | --- | --- | --- |
| Mediator model (DV: Blatant humanness rating) | | | | | |
| Peer gender conformity (path a) | 3.076 | 3.406 | 0.903 | .367 | −3.618, 9.770 |
| Age group | 0.282 | 0.775 | 0.364 | .716 | −1.241, 1.804 |
| Peer gender conformity x age group | −4.868 | 1.213 | −4.014 | < .001 | −7.252, −2.484 |
| DV model (DV: Bullying tendency) | | | | | |
| Peer gender conformity (path c’) | 0.218 | 0.044 | 4.953 | < .001 | 0.132, 0.305 |
| Blatant humanness rating (path b) | −0.004 | 0.001 | −4.100 | < .001 | −0.006, −0.002 |
| Participant gender | −0.040 | 0.050 | −0.801 | .424 | −0.139, 0.059 |
| Participant gender x peer gender conformity | −0.092 | 0.060 | −1.554 | .121 | −0.209, 0.025 |
|  | Estimate | | 95% CI | | |
| Index of Moderated Mediation | .**021** | | **.008, .037** | | |

**Remarks**. Peer gender conformity (0 = gender conforming; 1 = gender nonconforming); Participant gender (0 = boy; 1 = girl); *b* = unstandardized regression coefficients, *SE* = standard error; CI = confidence interval; Bootstrap sample size = 10,000.

**Model 2: Mediation via frequency proportion (*N* = 427)**

| Predictor variables | *b* | *SE* | *t* | *p* | 95% CI |
| --- | --- | --- | --- | --- | --- |
| Mediator model (DV: Frequency proportion) | | | | | |
| Peer gender conformity (path a) | 0.004 | 0.004 | 1.079 | .281 | −0.003, 0.011 |
| Age group | 0.005 | 0.001 | 5.425 | < .001 | 0.003, 0.006 |
| Peer gender conformity x age group | −0.002 | 0.001 | −1.427 | .154 | −0.005, 0.001 |
| DV model (DV: Bullying tendency) | | | | | |
| Peer gender conformity (path c’) | 0.289 | 0.045 | 6.426 | < .001 | 0.201, 0.378 |
| Frequency proportion (path b) | 1.057 | 1.015 | 1.042 | .298 | −0.938, 3.053 |
| Participant gender | −0.063 | 0.052 | −1.214 | .226 | −0.164, 0.039 |
| Participant gender x peer gender conformity | −0.115 | 0.063 | −1.826 | .069 | −0.239, 0.009 |
|  | Estimate | | 95% CI | | |
| Index of Moderated Mediation | −.002 | | −.009, .002 | | |

**Remarks**. Peer gender conformity (0 = gender conforming; 1 = gender nonconforming); Participant gender (0 = boy; 1 = girl); *b* = unstandardized regression coefficients, *SE* = standard error; CI = confidence interval; Bootstrap sample size = 10,000.

**Model 3: Mediation via diversity proportion (*N* = 423)**

| Predictor variables | *b* | *SE* | *t* | *p* | 95% CI |
| --- | --- | --- | --- | --- | --- |
| Mediator model (DV: Diversity proportion) | | | | | |
| Peer gender conformity (path a) | −0.001 | 0.002 | −0.459 | .647 | −0.006, 0.004 |
| Age group | 0.003 | 0.001 | 4.669 | < .001 | 0.002, 0.004 |
| Peer gender conformity x age group | 0.000 | 0.001 | −0.517 | .605 | −0.002, 0.001 |
| DV model (DV: Bullying tendency) | | | | | |
| Peer gender conformity (path c’) | 0.292 | 0.045 | 6.525 | < .001 | 0.204, 0.380 |
| Diversity proportion (path b) | 0.739 | 1.611 | 0.459 | .647 | −2.428, 3.905 |
| Participant gender | −0.080 | 0.051 | −1.550 | .122 | −0.181, 0.021 |
| Participant gender x peer gender conformity | −0.147 | 0.063 | −2.351 | .019 | −0.270, −0.024 |
|  | Estimate | | 95% CI | | |
| Index of Moderated Mediation | .000 | | −.004, .003 | | |

**Remarks**. Peer gender conformity (0 = gender conforming; 1 = gender nonconforming); Participant gender (0 = boy; 1 = girl); *b* = unstandardized regression coefficients, *SE* = standard error; CI = confidence interval; Bootstrap sample size = 10,000.

1. **Testing of multilevel moderated mediation models for girl peer condition**

**Model 1: Mediation via blatant humanness rating (*N* = 432)**

| Predictor variables | *b* | *SE* | *t* | *p* | 95% CI |
| --- | --- | --- | --- | --- | --- |
| Mediator model (DV: Blatant humanness rating) | | | | | |
| Peer gender conformity (path a) | −0.970 | 3.400 | −0.285 | .776 | −7.652, 5.712 |
| Age group | 1.001 | 0.740 | 1.354 | .177 | −0.453, 2.456 |
| Peer gender conformity x age group | −3.199 | 1.211 | −2.642 | .009 | −5.578, −0.819 |
| DV model (DV: Bullying tendency) | | | | | |
| Peer gender conformity (path c’) | 0.119 | 0.043 | 2.771 | .006 | 0.035, 0.204 |
| Blatant humanness rating (path b) | −0.003 | 0.001 | −2.984 | .003 | −0.005, −0.001 |
| Participant gender | −0.090 | 0.052 | −1.729 | .085 | −0.192, 0.012 |
| Participant gender x peer gender conformity | 0.032 | 0.059 | 0.544 | .587 | −0.084, 0.148 |
|  | Estimate | | 95% CI | | |
| Index of Moderated Mediation | **.010** | | **.002, .021** | | |

**Remarks**. Peer gender conformity (0 = gender conforming; 1 = gender nonconforming); Participant gender (0 = boy; 1 = girl); *b* = unstandardized regression coefficients, *SE* = standard error; CI = confidence interval; Bootstrap sample size = 10,000.

**Model 2: Mediation via frequency proportion (*N* = 427)**

| Predictor variables | *b* | *SE* | *t* | *p* | 95% CI |
| --- | --- | --- | --- | --- | --- |
| Mediator model (DV: Frequency proportion) | | | | | |
| Peer gender conformity (path a) | 0.001 | 0.003 | 0.296 | .768 | −0.006, 0.008 |
| Age group | 0.006 | 0.001 | 6.337 | < .001 | 0.004, 0.007 |
| Peer gender conformity x age group | −0.002 | 0.001 | −1.403 | .161 | −0.004, 0.001 |
| DV model (DV: Bullying tendency) | | | | | |
| Peer gender conformity (path c’) | 0.137 | 0.045 | 3.072 | .002 | 0.050, 0.225 |
| Frequency proportion (path b) | −0.315 | 1.116 | −0.282 | .778 | −2.509, 1.879 |
| Participant gender | −0.119 | 0.054 | −2.206 | .028 | −0.225, −0.013 |
| Participant gender x peer gender conformity | 0.059 | 0.062 | 0.947 | .344 | −0.064, 0.182 |
|  | Estimate | | 95% CI | | |
| Index of Moderated Mediation | .001 | | −.004, .006 | | |

**Remarks**. Peer gender conformity (0 = gender conforming; 1 = gender nonconforming); Participant gender (0 = boy; 1 = girl); *b* = unstandardized regression coefficients, *SE* = standard error; CI = confidence interval; Bootstrap sample size = 10,000.

**Model 3: Mediation via diversity proportion (*N* = 423)**

| Predictor variables | *b* | *SE* | *t* | *p* | 95% CI |
| --- | --- | --- | --- | --- | --- |
| Mediator model (DV: Diversity proportion) | | | | | |
| Peer gender conformity (path a) | 0.006 | 0.002 | 2.821 | .005 | 0.002, 0.010 |
| Age group | 0.004 | 0.001 | 6.715 | < .001 | 0.003, 0.005 |
| Peer gender conformity x age group | −0.003 | 0.001 | −3.653 | < .001 | −0.004, −0.001 |
| DV model (DV: Bullying tendency) | | | | | |
| Peer gender conformity (path c’) | 0.137 | 0.045 | 3.053 | .002 | 0.049, 0.226 |
| Diversity proportion (path b) | 0.992 | 1.749 | 0.567 | .571 | −2.447, 4.430 |
| Participant gender | −0.123 | 0.054 | −2.282 | .023 | −0.229, −0.017 |
| Participant gender x peer gender conformity | 0.053 | 0.063 | 0.844 | .399 | −0.071, 0.177 |
|  | Estimate | | 95% CI | | |
| Index of Moderated Mediation | −.003 | | −.014, .007 | | |

**Remarks**. Peer gender conformity (0 = gender conforming; 1 = gender nonconforming); Participant gender (0 = boy; 1 = girl); *b* = unstandardized regression coefficients, *SE* = standard error; CI = confidence interval; Bootstrap sample size = 10,000.

1. **Testing of multilevel moderated mediation models by age group**

In boy peer condition and in girl peer condition, the index of moderated mediation was significant in the model with blatant humanness rating as mediator, whereas the index was non-significant in other models with frequency proportion or diversity proportion as mediators. To further investigate the significant moderated mediation effect, mediation analyses with blatant humanness rating as the mediator across different age group levels within each peer gender condition were conducted.

**Model 1:** **Mediation via blatant humanness rating by age group for boy peer condition**

| Predictor variables | *b* | *SE* | *t* | *p* | 95% CI |
| --- | --- | --- | --- | --- | --- |
| **5-6y age group (*N* = 99)** | | | | | |
| Mediator model (DV: Blatant humanness rating) | | | | | |
| Peer gender conformity (path a) | 0.444 | 2.866 | 0.155 | .877 | −5.243, 6.132 |
| DV model (DV: Bullying tendency) | | | | | |
| Peer gender conformity (path c’) | 0.205 | 0.090 | 2.267 | .026 | 0.026, 0.384 |
| Blatant humanness rating (path b) | 0.000 | 0.002 | −0.115 | .909 | −0.005, 0.004 |
| Participant gender | −0.209 | 0.124 | −1.677 | .097 | −0.456, 0.038 |
| Participant gender x peer gender conformity | −0.216 | 0.125 | −1.724 | .088 | −0.465, 0.033 |
| **7-8y age group (*N* = 103)** |  |  |  |  |  |
| Mediator model (DV: Blatant humanness rating) | | | | | |
| Peer gender conformity (path a) | −8.476 | 2.864 | −2.960 | .004 | −14.156, −2.800 |
| DV model (DV: Bullying tendency) |  |  |  |  |  |
| Peer gender conformity (path c’) | 0.092 | 0.093 | 0.997 | .321 | −0.091, 0.276 |
| Blatant humanness rating (path b) | −0.003 | 0.002 | −1.296 | .198 | −0.007, 0.002 |
| Participant gender | 0.089 | 0.105 | 0.842 | .402 | −0.120, 0.298 |
| Participant gender x peer gender conformity | 0.007 | 0.125 | 0.054 | .957 | −0.242, 0.256 |
| **9-10y age group (*N* = 112)** |  |  |  |  |  |
| Mediator model (DV: Blatant humanness rating) | | | | | |
| Peer gender conformity (path a) | −14.116 | 2.677 | −5.274 | < .001 | −19.420, −8.812 |
| DV model (DV: Bullying tendency) | |  |  |  |  |
| Peer gender conformity (path c’) | 0.222 | 0.085 | 2.608 | .010 | 0.053, 0.391 |
| Blatant humanness rating (path b) | −0.005 | 0.002 | −2.453 | .016 | −0.009, −0.001 |
| Participant gender | −0.075 | 0.085 | −0.878 | .382 | −0.243, 0.094 |
| Participant gender x peer gender conformity | −0.041 | 0.114 | −0.362 | .718 | −0.267, 0.185 |
| **11-12y age group (*N* = 118)** |  |  |  |  |  |
| Mediator model (DV: Blatant humanness rating) | | | | | |
| Peer gender conformity (path a) | −14.229 | 2.468 | −5.764 | < .001 | −19.117, −9.340 |
| DV model (DV: Bullying tendency) | |  |  |  |  |
| Peer gender conformity (path c’) | 0.308 | 0.086 | 3.598 | < .001 | 0.139, 0.478 |
| Blatant humanness rating (path b) | −0.006 | 0.002 | −3.035 | .003 | −0.011, −0.002 |
| Participant gender | 0.011 | 0.085 | 0.133 | .895 | −0.157, 0.180 |
| Participant gender x peer gender conformity | −0.131 | 0.112 | −1.170 | .245 | −0.354, 0.091 |
| Indirect effects | *b* | *SE* | *Z* | *p* | 95% CI |
| 5-6y age group | 0.000 | 0.006 | −0.018 | .986 | −0.015, 0.014 |
| 7-8y age group | 0.024 | 0.021 | 1.134 | .257 | −0.012, 0.070 |
| 9-10y age group | 0.070 | 0.032 | 2.192 | **.028** | **0.012, 0.137** |
| 11-12y age group | 0.091 | 0.034 | 2.655 | **.008** | **0.029, 0.162** |

**Remarks**. Peer gender conformity (0 = gender conforming; 1 = gender nonconforming); Participant gender (0 = boy; 1 = girl); *b* = unstandardized regression coefficients, *SE* = standard error; CI = confidence interval; Bootstrap sample size = 10,000.

**Model 2:** **Mediation via blatant humanness rating by age group for girl peer condition**

| Predictor variables | *b* | *SE* | *t* | *p* | 95% CI |
| --- | --- | --- | --- | --- | --- |
| **5-6y age group (*N* = 99)** | | | | | |
| Mediator model (DV: Blatant humanness rating) | | | | | |
| Peer gender conformity (path a) | −2.101 | 2.824 | −0.744 | .459 | −7.704, 3.502 |
| DV model (DV: Bullying tendency) | | | | | |
| Peer gender conformity (path c’) | 0.197 | 0.118 | 1.672 | .098 | −0.037, 0.431 |
| Blatant humanness rating (path b) | −0.003 | 0.003 | −1.030 | .306 | −0.009, 0.003 |
| Participant gender | −0.352 | 0.130 | −2.700 | .008 | −0.610, −0.093 |
| Participant gender x peer gender conformity | 0.049 | 0.162 | 0.302 | .763 | −0.273, 0.371 |
| **7-8y age group (*N* = 103)** |  |  |  |  |  |
| Mediator model (DV: Blatant humanness rating) | | | | | |
| Peer gender conformity (path a) | −10.524 | 3.046 | −3.455 | < .001 | −16.567, −4.482 |
| DV model (DV: Bullying tendency) |  |  |  |  |  |
| Peer gender conformity (path c’) | 0.056 | 0.092 | 0.607 | .545 | −0.126, 0.238 |
| Blatant humanness rating (path b) | 0.000 | 0.002 | 0.006 | .995 | −0.004, 0.004 |
| Participant gender | 0.078 | 0.113 | 0.691 | .491 | −0.147, 0.303 |
| Participant gender x peer gender conformity | 0.072 | 0.122 | 0.587 | .558 | −0.171, 0.314 |
| **9-10y age group (*N* = 112)** |  |  |  |  |  |
| Mediator model (DV: Blatant humanness rating) | | | | | |
| Peer gender conformity (path a) | −10.241 | 2.495 | −4.105 | < .001 | −15.184, −5.298 |
| DV model (DV: Bullying tendency) |  |  |  |  |  |
| Peer gender conformity (path c’) | 0.096 | 0.059 | 1.627 | .107 | −0.021, 0.214 |
| Blatant humanness rating (path b) | −0.006 | 0.002 | −3.516 | < .001 | −0.009, −0.003 |
| Participant gender | −0.082 | 0.077 | −1.061 | .291 | −0.235, 0.071 |
| Participant gender x peer gender conformity | 0.024 | 0.085 | 0.287 | .775 | −0.143, 0.192 |
| **11-12y age group (*N* = 118)** |  |  |  |  |  |
| Mediator model (DV: Blatant humanness rating) | | | | | |
| Peer gender conformity (path a) | −13.051 | 2.500 | −5.221 | < .001 | −18.001, −8.101 |
| DV model (DV: Bullying tendency) | |  |  |  |  |
| Peer gender conformity (path c’) | 0.120 | 0.076 | 1.574 | .118 | −0.031, 0.270 |
| Blatant humanness rating (path b) | −0.005 | 0.002 | −2.847 | .005 | −0.009, −0.002 |
| Participant gender | −0.040 | 0.083 | −0.474 | .636 | −0.205, 0.126 |
| Participant gender x peer gender conformity | −0.041 | 0.102 | −0.398 | .692 | −0.243, 0.162 |
| Indirect effects | *b* | *SE* | *Z* | *p* | 95% CI |
| 5-6y age group | 0.006 | 0.013 | 0.474 | .636 | −0.016, 0.038 |
| 7-8y age group | 0.000 | 0.022 | −0.006 | .995 | −0.045, 0.043 |
| 9-10y age group | 0.058 | 0.022 | 2.626 | **.009** | **0.020, 0.106** |
| 11-12y age group | 0.070 | 0.029 | 2.465 | **.014** | **0.019, 0.131** |

**Remarks**. Peer gender conformity (0 = gender conforming; 1 = gender nonconforming); Participant gender (0 = boy; 1 = girl); *b* = unstandardized regression coefficients, *SE* = standard error; CI = confidence interval; Bootstrap sample size = 10,000.

Consistent with the original models, significant indirect effects via blatant humanness rating were observed only in the 9-10y and 11-12y age groups in boy peer condition as well as in girl peer condition.

**References**

Cohen, J. (1988). *Statistical Power Analysis for the Behavioral Sciences* (2nd ed.). L. Erlbaum Associates.

Wobbrock, J. O., Findlater, L., Gergle, D., & Higgins, J. J. (2011, May). The aligned rank transform for nonparametric factorial analyses using only anova procedures. In *Proceedings of the SIGCHI Conference on Human Factors in Computing Systems* (pp. 143-146). <https://doi.org/10.1145/1978942.1978963>
